# Reverse-Engineering Post-Transcriptional Regulation of Gap Genes in *Drosophila melanogaster*

**DOI:** 10.1371/journal.pcbi.1003281

**Published:** 2013-10-31

**Authors:** Kolja Becker, Eva Balsa-Canto, Damjan Cicin-Sain, Astrid Hoermann, Hilde Janssens, Julio R. Banga, Johannes Jaeger

**Affiliations:** 1 EMBL/CRG Research Unit in Systems Biology, Centre de Regulació Genòmica, and Universitat Pombeu Fabra (UPF), Barcelona, Spain; 2 Institute of Genetics, Johannes Gutenberg University, Mainz, Germany; 3 Bioprocess Engineering Group, IIM-CSIC, Vigo, Spain; Princeton University, United States of America

## Abstract

Systems biology proceeds through repeated cycles of experiment and modeling. One way to implement this is reverse engineering, where models are fit to data to infer and analyse regulatory mechanisms. This requires rigorous methods to determine whether model parameters can be properly identified. Applying such methods in a complex biological context remains challenging. We use reverse engineering to study post-transcriptional regulation in pattern formation. As a case study, we analyse expression of the gap genes *Krüppel*, *knirps*, and *giant* in *Drosophila melanogaster*. We use detailed, quantitative datasets of gap gene mRNA and protein expression to solve and fit a model of post-transcriptional regulation, and establish its structural and practical identifiability. Our results demonstrate that post-transcriptional regulation is not required for patterning in this system, but is necessary for proper control of protein levels. Our work demonstrates that the uniqueness and specificity of a fitted model can be rigorously determined in the context of spatio-temporal pattern formation. This greatly increases the potential of reverse engineering for the study of development and other, similarly complex, biological processes.

## Author Summary

The analysis of pattern-forming gene networks is largely focussed on transcriptional regulation. However, post-transcriptional events, such as translation and regulation of protein stability also play important roles in the establishment of protein expression patterns and levels. In this study, we use a reverse-engineering approach—fitting mathematical models to quantitative expression data—to analyse post-transcriptional regulation of the *Drosophila* gap genes *Krüppel*, *knirps* and *giant*, involved in segment determination during early embryogenesis. Rigorous fitting requires us to establish whether our models provide a robust and unique solution. We demonstrate, for the first time, that this can be done in the context of a complex spatio-temporal regulatory system. This is an important methodological advance for reverse-engineering developmental processes. Our results indicate that post-transcriptional regulation is not required for pattern formation, but *is* necessary for proper regulation of gap protein levels. Specifically, we predict that translation rates must be tuned for rapid early accumulation, and protein stability must be increased for persistence of high protein levels at late stages of gap gene expression.

## Introduction

**Figure 1:**
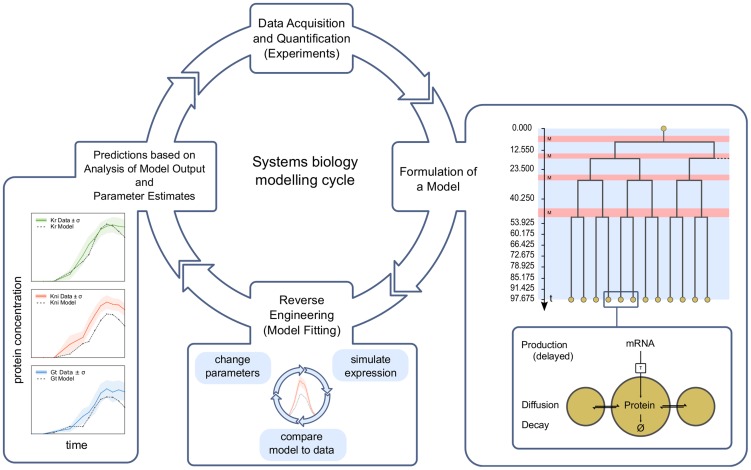
The systems biology modeling cycle. This cycle illustrates the interplay of experiment and modeling in modern systems biology (adapted from [26]). Expression data are acquired and quantified. A model is formulated based on a regulatory hypothesis intended to explain the observed expression patterns. The model is solved and fit to data (reverse engineering). Model output and parameter values are then analysed to yield predictions and interpretations of the biological data. If necessary, the process is repeated—acquiring new data and improving the model—until a satisfactory explanation of the observed phenomena is achieved. Model fits are shown on the left. The panel describing the model depicts the processes of protein production, diffusion, and decay within and between nuclei (energids; lower panel). The upper panel shows the mitotic schedule (M: mitosis, red; otherwise: interphase, blue background), with those time points indicated for which we have data. See text for details.

Systems biology is characterised by the tight integration of experiments and computational modeling. One way to achieve such integration is through reverse-engineering approaches, where dynamical models of regulatory or biochemical reaction networks are fit to quantitative data [1]–[9]. Reverse engineering has been successfully used for systems analysis in many contexts, from microbial metabolic, signaling and regulatory networks (see, for example, [10]–[20]) to pattern-forming developmental processes in animals (e.g. [21]–[25]). The approach is illustrated by the systems biology modeling cycle shown in Figure 1 (adapted from [26]). As a first step, a mathematical model is formulated that incorporates the basic assumptions and hypotheses we have about the regulatory process under study. The model is then tested by fitting it to metabolic or expression data. This is achieved by repeatedly altering its parameters and selecting suitable (mainly better-fitting) solutions. A successful fit will yield a unique set of parameter estimates that cause the model to reproduce the data accurately. In this case, model output and estimated parameter values can be analysed to gain biological insight. For instance, regulatory parameters contain information on the strength and type of regulatory interactions in a network. If the model fails to produce a unique solution—predicting a large set of variant networks instead—it is underdetermined and more data need to be collected. If the model cannot fit the data, the underlying hypothesis needs to be adjusted, or additional mechanisms and factors need to be incorporated. Successive model-fitting/data-acquisition cycles yield an increasingly accurate quantitative picture of the underlying regulatory network.

While this approach has great potential for the investigation of complex biological regulatory systems (e.g. [27]), it also harbors many significant and non-trivial challenges. One of those is that it is often difficult to decide what kind of data, and what kind of model are needed to enable a successful fit. Another challenge is to analyse whether a given solution is indeed specific and reliable. There are a number of mathematical methods designed to establish whether a reverse-engineering problem is well posed—in other words, whether it is able to produce a unique and consistent solution [5], [7], [28]–[30]. First, structural (or *a priori*) parameter identifiability analysis can be used to examine whether the problem has a non-trivial solution at all [31]–[33]. Second, practical (or *a posteriori*) parameter identifiability analysis tells us whether estimated parameter values are significant and reliable [31], [34]–[36]. Finally, methods for optimal experimental design are employed to determine what kind of measurements (for which regulatory factors and which time points, for example) would improve the quality of the fit most significantly [5], [37]–[40].

So far, unfortunately, the application of these powerful methods to gain specific and novel biological insights has been limited. This is due both to the complexity of most real-world biological regulatory systems and the nature of the data used in reverse-engineering studies. Most of these studies use models based on large systems of coupled non-linear differential equations. This makes it challenging to apply structural identifiability analysis. Moreover, model fitting is generally computationally intensive due to the significant number of parameters to be estimated. This renders rigorous practical identifiability analysis extremely time consuming. And finally, high-throughput datasets used for model fitting often exhibit high levels of measurement error, combined with low numbers of replicates. Under these circumstances, it is difficult to accurately assess data variance, which is required for both practical identifiability analysis and optimal experimental design. For all these reasons, reverse-engineering studies often proceed on an empirical basis, without being able to rigorously establish parameter identifiability or the suitability of the datasets and models used.

Here, we present a reverse-engineering study which combines model fitting by global optimisation strategies with rigorous structural and practical identifiability analysis. We apply this methodology to a complex regulatory problem: the dynamics of spatio-temporal pattern formation in the early embryo of the vinegar fly *Drosophila melanogaster*. The biological question we are addressing is the importance of post-transcriptional regulation in animal development. While many studies of pattern formation focus on differential transcriptional regulation of genes (e.g. [41], [42]), other levels of expression control—such as regulated RNA splicing, processing, translational regulation, or regulated stability and degradation of gene products—cannot be ignored [43]. There is increasing evidence that protein levels do not generally match those of their respective mRNAs [44]–[46], and many protein expression patterns do not even coincide with the timing and localisation of mRNA transcription [47], [48]. These discrepancies are due (at least in part) to control at the level of protein translation. Indeed, some of the earliest studies of translational control were carried out in *Drosophila* (reviewed in [48]). A number of pioneering studies examined the effect of translational repression on maternal morphogen gradients, such as those formed by the protein products of the maternal genes *hunchback (hb)* and *caudal (cad)*. mRNAs derived from those genes are distributed uniformly while their proteins form steep concentration gradients with antero-posterior polarity [49]–[54]. More recently, systems-level studies indicate that such post-transcriptional regulation is widespread and of general importance. Protein expression levels in yeast cannot be predicted from mRNA concentrations alone [55], and a similar lack of correlation between mRNA and protein is observed in systems as different as the minimal bacterium *Mycoplasma pneumoniae* [47] and mammalian cell lines [46]. Therefore, post-transcriptional regulatory mechanisms must be incorporated in a systems-level understanding of cellular and organismal function.

In this study, we investigate the role of post-transcriptional regulation within the context of a well established experimental model system: the gap genes involved in segment determination during the blastoderm stage of early *Drosophila* development (reviewed in [56]). Since the relevance of post-transcriptional regulation for maternal *hb* expression is well established (see above, and [52], [53]), we will focus on the remaining three trunk gap genes: *Krüppel (Kr)*, *knirps (kni)*, and *giant (gt)*. All these genes encode transcription factors, and are expressed in broad, overlapping domains along the embryo's antero-posterior (A–P) axis. Gap genes respond to activating transcriptional regulatory inputs from long-range maternal morphogen gradients—such as Bicoid (Bcd), Hb, and Caudal (Cad)—as well as repressive inputs from the terminal gap genes *tailless (tll)* and *huckebein (hkb)*. In addition, there is extensive repressive gap gene cross-regulation, which is required for the correct dynamic positioning, maintenance, and sharpening of each gap gene expression domain.

The advantages of using the gap gene network for our case study are twofold. The first advantage is that gap gene patterning is relatively simple and tractable compared to other developmental processes. It essentially occurs in one dimension, along the A–P axis of the embryo. No significant tissue rearrangements or growth are involved. Diffusion is not yet limited by cell membranes as the embryo is still syncytial at this stage. In addition, all three genes considered here have a very compact structure, with only one or two short introns, and none of them exhibits any sign of alternative splicing. The second advantage is that the gap gene system is exceptionally well understood. All genes involved in segment determination have been identified and their interactions have been characterised at the genetic and molecular level (see [56] and references therein). More importantly, there exist extensive quantitative datasets (including accurate variance measurements) for spatio-temporal gap mRNA and protein expression [56]–[63]. These datasets have been used to fit a range of gene regulatory network models, analysis of which has led to many quantitative systems-level insights into the dynamic mechanisms underlying gap gene regulation [22]–[25], [36], [58], [64]–[66].

All these previously published models focus on transcriptional regulation of gap genes. They lump together transcriptional and post-transcriptional phases of gene regulation, and take into account only protein concentrations (not mRNA) as model observables. Therefore, these models implicitly assume that post-transcriptional regulation is not required to explain the patterns formed by gap genes. This assumption is not unreasonable, given the similarity of gap mRNA and protein patterns, and the fact that such simplified models can reproduce gap protein patterns to a high degree of accuracy and temporal resolution [22], [36]. Moreover, the experimental literature contains very little evidence or arguments for post-transcriptional regulation of *Kr*, *kni*, or *gt*. The only exception we could find is a paper by Gaul et al. [67], which invoked post-transcriptional regulation of *Kr* to explain the anterior displacement of its mRNA domain with respect to the protein pattern. This phenomenon was later shown to be due to the dynamic anterior shift of the central *Kr* domain [22]. However, absence of evidence is not evidence of absence. Therefore, it is necessary to put the hypothesis that post-transcriptional regulation is not required for gap gene patterning to a rigorous and quantitative test.

As mentioned above, we test this hypothesis using a reverse-engineering approach. This is achieved by formulating a model, which incorporates the simple assumption that gap protein patterns reflect those of their respective mRNAs a given amount of time earlier in development (plus a small contribution by gap protein diffusion; see Figure 1, right-hand panel). Since we do not consider gap gene cross-regulation, we can model *Kr*, *kni*, and *gt* separately. Each model is then fitted to protein expression data, using mRNA patterns as external inputs, or boundary conditions. If our models are able to reproduce gap protein patterns correctly, we can conclude that no post-transcriptional regulation is required for the expression of the gap genes considered here. If our models fail to fit, however, we will be able to identify those expression features that do rely on post-transcriptional regulatory processes.

Our model consists of the following system of ordinary differential equations, representing the change of gap protein concentration 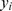 over time 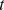 in a row of dividing nuclei 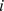 along the A–P axis of the embryo:
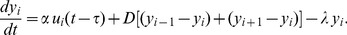
(1)Here, the dependence of protein on mRNA patterns is linear. 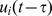 is a time-delayed external input representing mRNA concentration 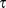 minutes ago. We obtain 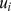 for arbitrary time points 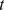 by linear interpolation from measured expression data points. Transcription is paused during mitosis, when chromosomes are condensed. Therefore, the production term is set to zero whenever 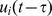 falls into a mitotic period (see Materials and Methods for time schedule). 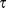 is a production delay which summarises the time from initiation of transcription (when transcripts become detectable by our staining methods) to the appearance of the resulting protein. It includes contributions from the completion of transcription, RNA splicing and processing, nuclear export, and translation. 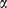 is the rate of protein production from mRNA. 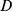 is the rate of gap protein diffusion among neighboring nuclei 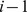, 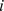, and 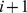. It depends on the square of the distance between nuclei which is halved upon each nuclear division [64]. 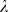 is the protein degradation rate.

After establishing the structural identifiability of our parameters, we need to estimate 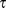 (production delay), 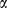 (production rate), 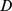 (diffusion rate), and 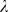 (degradation rate) by fitting the model to data. This is achieved by minimising the weighted sum of squared differences between experimentally measured (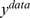) and modeled protein expression values (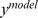) for each nucleus 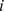 and time point 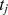 for which we have data:
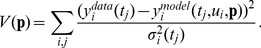
(2)

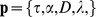
 is the parameter vector to be estimated. Weights 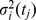 are given by measured variances for each data point. Equation (2) represents a weighted least squares (WLS) problem, which we solve using global optimisation methods as described below. The reliability and accuracy of the resulting parameter estimates are then analysed using practical parameter identifiability analysis.

Using the reverse-engineering approach described above, we have obtained fitted models and parameter estimates for post-transcriptional regulation of *Kr*, *kni*, and *gt*. Identifiability analysis shows that our fitting results and parameter estimates are robust and specific. They yield values for rate parameters which are biologically plausible and informative with regard to the time scale and diffusive properties of gap gene patterning. Our fits reveal that post-transcriptional regulation is not required for the correct timing and positioning of gap protein domain boundaries. They do suggest, however, that post-transcriptional regulation *is* required for the accurate control of gap protein levels, implying some temporal regulation of translational efficiency, and/or protein stability. Specifically, our models predict an early boost in translational efficiency, plus a general stabilisation of gap protein products towards the end of the blastoderm stage.

## Results

### Quantitative Gap Gene mRNA Expression Data

**Figure 2:**
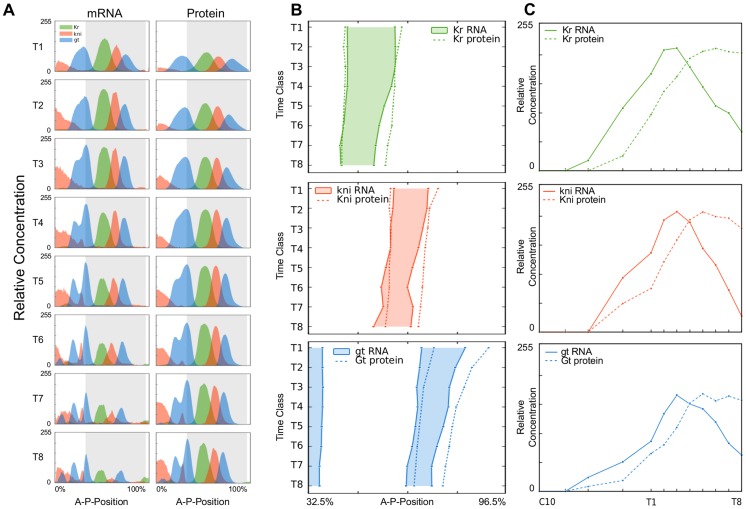
Comparison of gap gene mRNA and protein expression patterns. (A) Time series showing integrated one-dimensional expression patterns of gap gene mRNA (left column) and protein (right column) along the A–P axis in cleavage cycle C14A (time classes T1–T8). *Kr* is shown in green, *kni* in red, and *gt* in blue. X-axes represent A–P position in %, where 0% corresponds to the anterior pole of the embryo. Y-axes represent mRNA and protein concentrations in relative units. Grey background indicates the region displayed in (B). (B) Space-time plots indicating domain boundary positions of the central *Kr* domain (top), the abdominal *kni* domain (middle), and the anterior and posterior domains of *gt* (bottom). Solid patterns indicate mRNA patterns, dashed lines protein. Time flows downwards. (C) Temporal dynamics of peak expression for the central *Kr* domain (top), the abdominal *kni* domain (middle), and the posterior *gt* domain (bottom). Solid lines indicate mRNA, dashed lines protein. Relative concentrations (as in (A)) are plotted against time.

We have created a quantitative mRNA expression dataset with high spatial and temporal resolution for the trunk gap genes *Krüppel (Kr)*, *knirps (kni)* and *giant (gt)*, which spans the entire duration of the blastoderm stage in the early embryo of *Drosophila melanogaster* (cleavage cycles C10–C14A; C14A is further subdivided into time classes T1–8). In contrast to previously published semi-quantitative gap gene mRNA data—based on colorimetric (enzymatic) staining protocols, wide-field microscopy, and an efficient but simple data processing pipeline [66], [68] —we used fluorescent staining protocols, confocal microscopy and fully quantitative data processing methods (see Materials and Methods, and [69]). This work extends a previously published fully quantitative expression dataset for gap gene mRNAs, which only covered the early part of the blastoderm stage (C10–C13) [58]. Our data consist of quantified time-series of gap gene mRNA expression (Figure 2; Supplementary Figures S1, S2, S3), which are equivalent and comparable in quality, as well as spatio-temporal range and resolution, to the comprehensive protein expression data available from the FlyEx database (http://urchin.spbcas.ru/flyex, [57], [59], [60]). This allows us, for the first time, to rigorously and accurately compare gap gene expression during the blastoderm stage at both mRNA and protein level.

Such a comparison between spatio-temporal gap mRNA and protein expression reveals that, to a first approximation, the mRNA patterns appear very similar to those observed for the corresponding proteins: all mRNA transcripts are expressed in broad, overlapping domains, whose relative timing and spatial arrangement with regard to each other strongly resemble that of gap protein domains (Figure 2A). In addition, the central, abdominal, and posterior mRNA domains of *Kr*, *kni*, and *gt* shift towards the anterior of the embryo over time (Figure 2B). The extent of this movement is on the same order of magnitude as the analogous shifts of the corresponding protein domains (Figure 2B, Supplementary Table S1) [22], [59], [66]. Since anterior domain boundaries generally move less far than posterior ones, domain width of both mRNA and protein domains decreases over time (Figure 2B, Supplementary Table S1) [59]. Based on these observations, we can conclude that expression dynamics of gap mRNA and protein domains are largely qualitatively equivalent with regard to each other.

However, if we examine the data more closely, two significant differences between mRNA and protein become apparent. First, boundary positions of mRNA domains—with the exception of the anterior *Kr* border—are displaced anteriorly compared to their corresponding protein domains (Figure 2B; Supplementary Table S1). This displacement is caused by the anterior shift of gap domains over time [22], [59]. The effect is substantially more pronounced for posterior domain borders than for anterior ones. It is associated with a generally slightly smaller width of mRNA domains compared to protein (Supplementary Table S1). Second, the timing of initial and maximum peak expression is delayed for protein compared to mRNA (Figure 2C). Delayed first appearance of protein versus mRNA patterns during the early blastoderm stage has been reported and quantified previously [58], [59]. Our data reveal a similar phenomenon in late-blastoderm expression dynamics: mRNA expression of all three gap genes peaks around 30 min before gastrulation (time class T3 in Figure 2C), while protein expression shows a maximum approximately 15–20 min later (time class T6–7). This obviously agrees with the fact that it takes time to export the mRNA from the nucleus, to process and translate it into protein. In addition, we detect a post-peak decrease in mRNA abundance that was not reported in an earlier PCR- and microarray-based analysis of pre-gastrulation gene expression with a lower temporal resolution than our data [70]. Interestingly, this trend is not reflected in levels of gap proteins, which only show a marginal decrease (if any) before the onset of gastrulation (see Figure 2C and also Supplementary Text S1).

In addition to our quantification of the timing and position of averaged gene expression patterns, we have also analysed the embryo-to-embryo variability of gap domain width as well as peak and boundary positions during the blastoderm stage. This had not been possible with our earlier, semi-quantitative dataset [66]. It has been reported previously that the precision of gap protein domain boundary positions increases over time due to cross-regulatory interactions [24], [25], [59]. Such a trend—although much less obvious—is also present in our mRNA data (Supplementary Figure S4). Noise levels in the mRNA data are generally higher than in protein patterns. Both *Kr* and *kni* (but not *gt*) show higher levels of variability in mRNA compared to protein data at a majority of sampled data points. Moreover, we observe a high level of fluctuations between time points in our mRNA data (for all three genes) indicating increased levels of experimental noise. This may be due to the harsher treatment of embryos for *in situ* hybridisation compared to antibody staining (see [58] for a more detailed discussion). Nevertheless, the overall trends are similar for mRNA and protein, as levels in variability of all measured expression features is generally lower at T6 than at earlier time points during C14 (Supplementary Figure S4). This indicates canalisation of development at two levels: first, protein patterns are generally more precise than mRNA domains at comparable stages, and second, mRNA precision—as is the case for protein—increases over time.

### Reverse Engineering: Structural Identifiability Analysis

The fact that mRNA and protein patterns of the gap genes *Kr*, *kni*, and *gt* are similar (yet not identical), and show a delay in dynamics with regard to each other, raises the non-trivial question whether protein patterns simply reflect earlier mRNA levels (with a small additional contribution by protein diffusion), or whether spatially and temporally specific post-transcriptional regulation is required to account for the observed distribution of proteins. We use a reverse-engineering approach to distinguish between these two alternative possibilities. In this approach, we test the hypothesis that no post-transcriptional regulation is required by fitting a simple dynamical model to data. This model incorporates the following assumptions (see Introduction): It includes time-delayed but linear production of protein from its mRNA, as well as protein diffusion and decay. It takes mRNA patterns as an input to predict protein expression. A good fit of the model to protein expression data would therefore favor absence of post-transcriptional regulation, while a failure to fit the data would point us to specific features of gap protein expression that require regulated nuclear export, splicing, or translation.

Our reverse-engineering approach can only give us quantitative and specific insights into the problem of post-transcriptional regulation if it is fit to data in a manner which is as rigorous and reproducible as possible. As a first step, this requires us to determine whether the model is formulated in a way such that the fitting procedure has a unique solution. Since our model is feed-forward and linear, this can be achieved using structural (or *a priori*) parameter identifiability analysis. This analysis is performed under an ideal scenario where noise-free time-continuous experimental data are assumed to be available, and the objective is to answer the question whether under those ideal conditions the parameters can be given unique values. There are three possible outcomes: (1) The model is structurally globally identifiable (s.g.i.) if all parameters 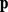 can be uniquely identified within a biologically meaningful region of parameter space, which we will call 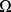 (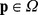). (2) The model is structurally locally identifiable (s.l.i.) if one or more parameters can be uniquely identified in a given neighborhood 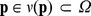, or (3) the model is not structurally identifiable, if neither of (1) or (2) apply.

Although several methods for the analysis of structural identifiability of linear models exist, the model described by equation (1) presents the peculiar challenge of incorporating a delay parameter within the input function 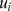 (the production term). In this scenario the Laplace transform (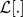) based method may be used to assess whether the s.g.i. condition holds (see [28] and references cited therein). The underlying idea is to verify whether a canonical form of the transfer matrix of the system 1 is unique. The basic steps are the following: the model (equation 1) is rewriten in matrix form and its Laplace transform is computed; the possibility of computing the transfer matrix is demonstrated by an invertibility conditon; the analytical canonical form of the transfer matrix is then obtained; symbolic manipulation is finally used to prove uniqueness of the transfer matrix. Details of our calculations can be found in Supplementary Text S2.

In the case of our model for post-transcriptional regulation (see Introduction, equation 1) structural identifiability analysis reveals that model parameters are globally identifiable in the realm of non-negative real numbers 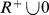 (which is expected given that rate and delay parameters cannot be less than zero). For this result to apply, the following conditions must be met by the experimental data: (1) some concentrations of mRNA and proteins must be non-zero in the interior of the model range at sampling times, and (2) protein data must be available for time points before or after mitosis (*i.e.* when the production rate is not zero; see Supplementary Text S2 for details). Both of these conditions are met in our data, and we conclude that our model parameters are globally structurally identifiable, *i. e.* our optimisation problem has a specific and unique solution.

### Reverse Engineering: Model Fitting

**Figure 3:**
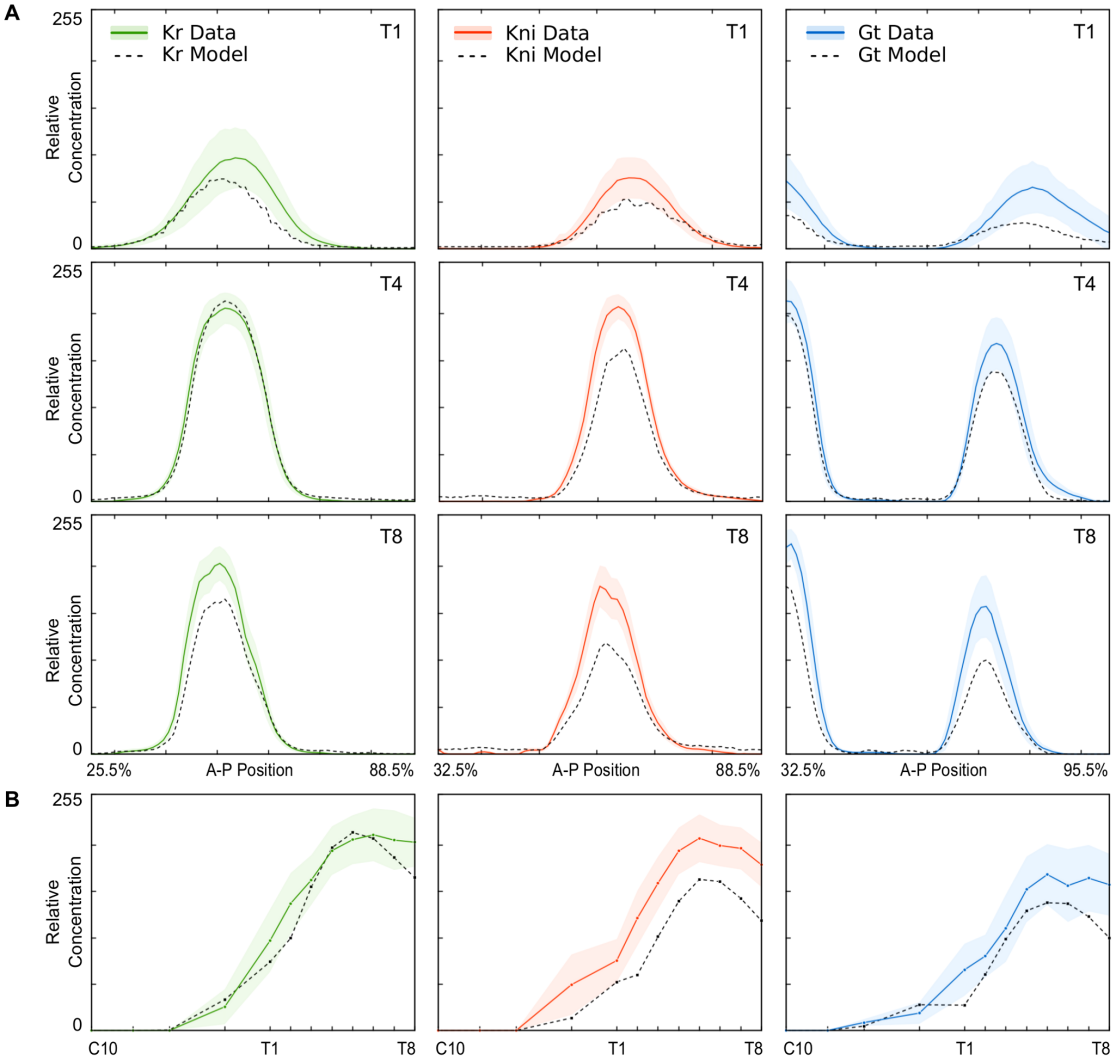
Comparison of model output and measured protein concentrations. (A) Spatial profiles of *Kr* (green), *kni* (red), and *gt* (blue) for early (T1), mid (T4), and late (T8) time classes during C14A. X-axes represent A–P position (in %), Y-axis show relative concentrations (as in Figure 2A). (B) Temporal dynamics of peak concentrations for the central *Kr* domain (left), the abdominal *kni* domain (centre), and the posterior *gt* domain (right). X-axes represent time, Y-axes show relative concentrations (as in Figure 2C). In all panels, model output is shown as a dashed black line; measured protein concentrations are shown as dark colored lines (mean) and lightly shaded background (standard deviations).

Next, we proceeded to fit our model to quantitative spatio-temporal protein expression data. This was done for *Kr*, *kni*, and *gt* separately by solving the model for each gene numerically, and minimising a weighted sum of squared differences between expression patterns predicted by the model and those measured by experiment (see equation 2). Minimisation of squared differences was achieved by two different global optimisation algorithms, based on parallel simulated annealing (pLSA), and an enhanced scatter search (eSS) method, respectively (see Materials and Methods for details and references). Both of these independent optimisation approaches resulted in equivalent model fits and parameter estimates (Figure 3; Table 1). In order to further corroborate the robust performance of our algorithms, we performed a systematic comparison of eSS with a number of standard global optimisation approaches. The results can be found in Supplementary Text S3. They indicate that different algorithms show significant differences in computational efficiency, but converge to very similar solutions.

**Table 1:** Comparison of residual scores and parameter estimates obtained from pLSA and eSS optimisation approaches.

	Kr_LSA_	Kr_eSS_	Kni_LSA_	Kni_eSS_	Gt_LSA_	Gt_eSS_
WLS	487.67	487.62	1960.10	1958.20	868.15	865.05
RMS	11.21	11.10	21.13	21.20	17.23	17.19
α	0.0970	0.0964	0.0783	0.0785	0.1107	0.1139
λ	0.0764	0.0756	0.0770	0.0772	0.1110	0.1139
D	0.0015	0.0015	0.0125	0.0126	0.0159	0.0180
τ	5.2953	5.1786	6.3083	6.3649	2.3900	2.6127

Scores and parameter estimates from two representative solutions (one for each optimisation method) are shown for *Kr*, *kni*, and *gt* models. WLS corresponds to the weighted least squares score 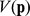 as defined in equation 2. RMS is the root-mean-square score as defined in equation 3. 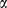 is the production rate, 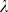 the decay rate, 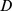 the diffusion rate, and 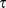 the production delay as defined in equation 1. LSA indicates scores and estimates from Lam Simulated Annealing, eSS scores and estimates from enhanced scatter search.

Fitting results differ slightly between genes. The best fit between model and data is obtained by Kr optimisation runs, with a root-mean-square (RMS) score of around 11.1–11.2 (Table 1; see Methods for a mathematical definition of RMS, which represents the average deviation of model from data for each data point). Although minor patterning defects can be observed at early stages (especially between C13 and T2) and expression levels disagree somewhat at the last time points (T7/T8, see below), model and data match to within the noise level of the data at intermediate times (Figure 3, left column).

Of all fitting solutions, Kni shows the largest overall deviation between model and data with a RMS score of 21.1–21.2 (Table 1). Nevertheless, position and shape of the Kni protein domain, as well as the temporal dynamics of expression, are reproduced correctly (Figure 3, centre column). In particular, the model shows peak expression at the correct stage, and agrees with data to high accuracy in non-expressing areas. In contrast, protein expression predicted by the model is consistently lower than measured within the area of the abdominal Kni domain. This discrepancy accounts for the large RMS value.

Expression patterns resulting from Gt optimization runs exhibit similar properties as those of Kni, with a slightly lower residual error (RMS around 17.2; see Table 1). Again, the timing, position, and shape of both Gt domains are reproduced quite accurately in the model, while predicted expression levels are generally too low (Figure 3, right column). The only noticeable positional defect is a slight anterior displacement of the posterior *gt* domain at early stages (up to T1).

Despite the problems with reproducing accurate levels of expression for Kni and Gt, all three models show initiation and build-up of gap proteins at the appropriate stages of development, and the qualitative shape of the temporal expression profile is reproduced correctly up to T6 (Figure 3B). In contrast, model and data disagree conspicuously at later time points (T7/8), since the model predicts rapidly diminishing concentrations of gap proteins before the onset of gastrulation. This downregulation is much weaker (Kni), or entirely absent (Kr, Gt) in the data.

In summary, our models capture the position, shape, and width of gap protein domains accurately. Minor deviations in these spatial expression features are only observed during earlier time points, when noise levels in the data are high. Temporal features of gap protein expression—such as initiation of expression, shifts in domain position, or the time point of maximum expression—are also reproduced correctly. However, our models fail to reproduce the exact levels of gap protein expression, as well as their downregulation towards the end of C14. These two specific failures of our model fits have interesting implications for our understanding of gap gene regulation (see Discussion).

### Reverse Engineering: Parameter Estimation and Analysis

Model fitting resulted in reproducible and biologically plausible estimates of parameter values for *Kr*, *kni*, and *gt*. We performed a number of independent optimisation runs for each model using both of our two alternative fitting strategies (100 runs of pLSA, 10 runs of eSS per model). Parameter estimates from different runs varied only minimally between solutions (for pLSA see Supplementary Figure S5), and estimated parameter values provided by either of the two alternative optimisation strategies agreed to high accuracy. This indicates that our parameter estimates are robust with regard to the choice of optimisation strategy. Table 1 shows parameter values from a representative optimisation run for each fitting approach.

Predicted values for the delay parameter 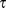 (see equation 1) require some more detailed attention, since such parameters are notoriously difficult to estimate. For this reason, we verified the validity of our estimates for 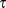 by the following numerical approach: we performed a series of fits for the *gt* model, with 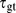 fixed to values between 0 and 8 minutes, including a particularly high-density sampling of 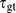 around the values predicted by optimisation (Supplementary Figure S6). These control fits show a minimum of the residual error which coincides precisely with the parameter values inferred from optimisation. This corroborates the reproducibility and accuracy of our approach.

Our parameter estimates are informative from a biological point of view, and yield experimentally testable predictions. First of all, we note that production and decay rates (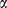 and 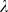, respectively) are well balanced in this system. Decay rates 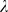 correspond to protein half lives of 9.1 (Kr), 9.0 (Kni), and 6.2 (Gt) minutes, indicating that gap proteins must be very unstable—to enable patterning at the extremely short time scale of gap gene expression dynamics. Gap protein diffusion is generally very low, especially in the case of Kr. Protein production delays (incorporating contributions of transcription, splicing, nuclear export, and translation; see Introduction) range between 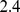 and 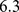 minutes. While the upper value is within the expected range (see Discussion) the former estimate is rather low, and may need further investigation (see also next section).

### Reverse Engineering: Practical Identifiability Analysis

While it is encouraging that independent optimisation runs and methods give consistent parameter estimates, it is necessary to test the reliability and accuracy of these estimates using practical (or *a posteriori*) parameter identifiability analysis (see Introduction). We have performed such an analysis using two complementary approaches.

One approach to the practical analysis of parameter identifiability is based on a geometrical interpretation of the ‘optimisation landscape’ given by the value of the weighted-least-squares cost function (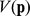, in equation (2)) [35], [36]. To illustrate this approach, we will assume a two-dimensional parameter space for simplicity. This results in a three-dimensional topography of the optimisation landscape, where minima lie in ‘troughs’ or ‘depressions’ of the contour determined by the cost function. The more shallow the trough in which a minimum lies, the more uncertain the parameter estimate, since changing parameter values around the optimum will lead to only a slight increase in the value of the cost function. It is possible to characterise the local surface of any optimisation landscape around a given minimum using linear approximations. This allows us to define an ellipsoidal confidence region around our minimum, resulting in estimates for the confidence intervals for each of our parameters.

If there is no correlation among parameters, the principal axes of the confidence ellipsoid will lie parallel to those of parameter space. Confidence intervals for parameters can then be calculated as the intersect of the ellipsoid with these axes. Correlations among parameters are detectable as an inclination between the ellipsoid's principal axes and the axes of parameter space. This makes it possible to calculate two distinct ranges: the dependent confidence interval is given by the intersection of the ellipsoid with a given parameter axis (as above), while the projection of the ellipsoid region onto the parameter axis specifies the independent confidence interval. Independent confidence intervals typically overestimate the uncertainty in parameters, while dependent confidence intervals underestimate it. If both confidence intervals turn out to be similar and small, a parameter can be considered well determined.

**Figure 4:**
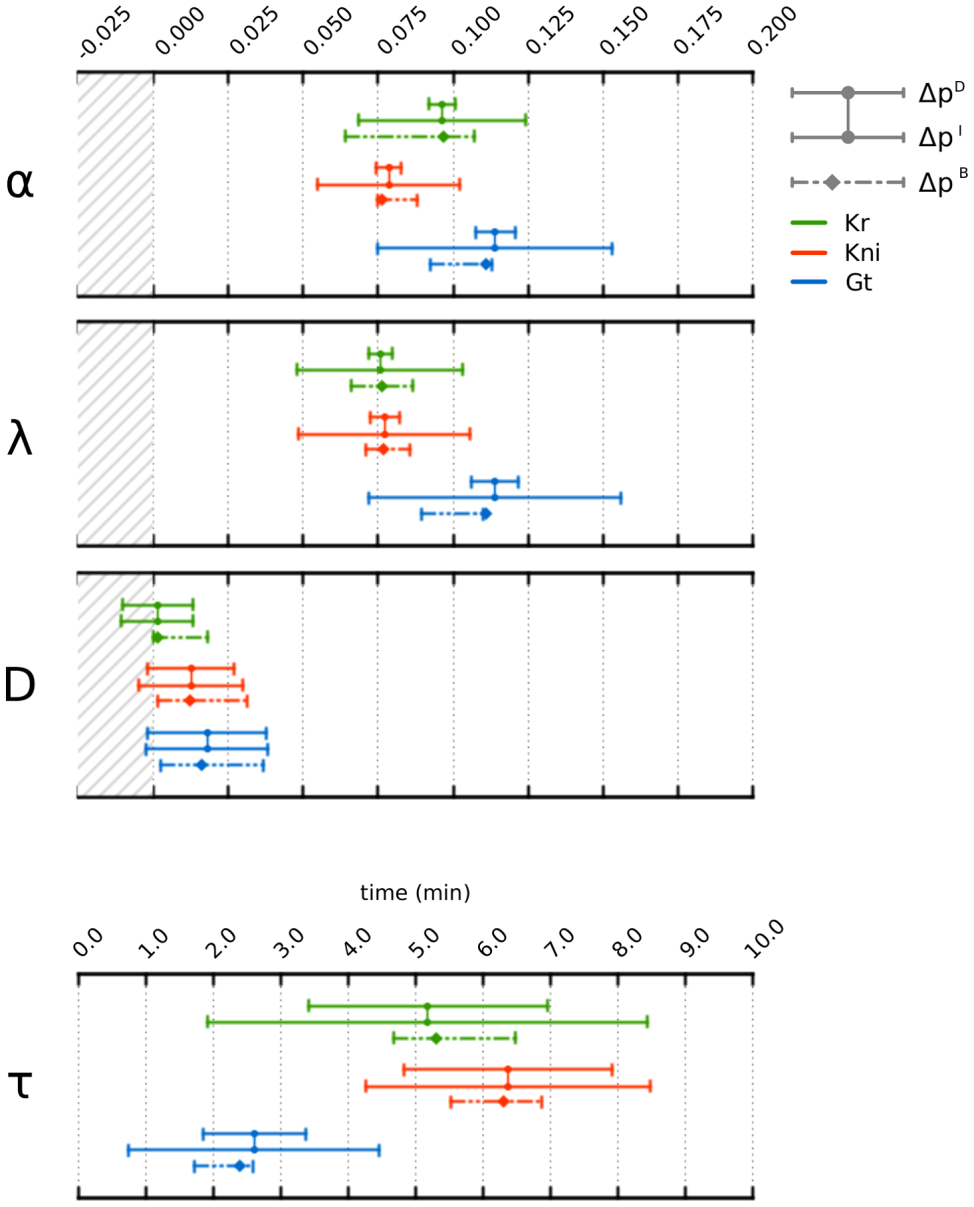
Confidence intervals for parameter estimates. This figure shows 95% confidence intervals for parameters 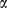 (production rate), 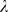 (decay rate), 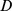 (diffusion rate), and 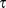 (production delay; see equation 1) for *Kr* (green), *kni* (red), and *gt* (blue). 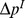 are independent, 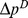 dependent intervals obtained from linear analysis (connected solid lines), 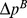 are intervals obtained from bootstrapping (dashed lines). Dots (on solid lines) represent eSS parameter estimates, diamonds (on dashed lines) those from SA. Striped grey background indicates parameter values that lie outside the search space limits used for optimisation. Note that only a subregion of the search space is shown in each panel (see Materials and Methods for values of search space limits).

Confidence intervals, as calculated by equations (4) and (5) (see Materials and Methods) are shown in Figure 4. Compared to the entire range of search space, confidence regions for all three rate parameters (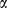, 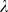, and 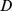; see equation (1)) are small in models of all three gap genes. Dependent and independent confidence intervals for 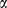 and 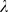 deviate significantly, suggesting strong mutual correlation among model parameters. This is not the case for 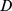, where independent and dependent confidence intervals are very similar. Note that the lower limits of some intervals for 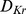 and 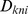 are negative, and therefore lie outside the allowed range of parameter values. This artifact results from the linear approximation of the optimisation landscape used in this method. Confidence intervals for delay parameters 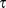 are larger compared to rate parameters. Nevertheless, they lie within a well confined and biologically plausible range (Figure 4). As for the case of 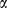 and 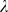 above, there appears to be a high degree of correlation for 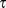 with other parameters in all three models.

**Figure 5:**
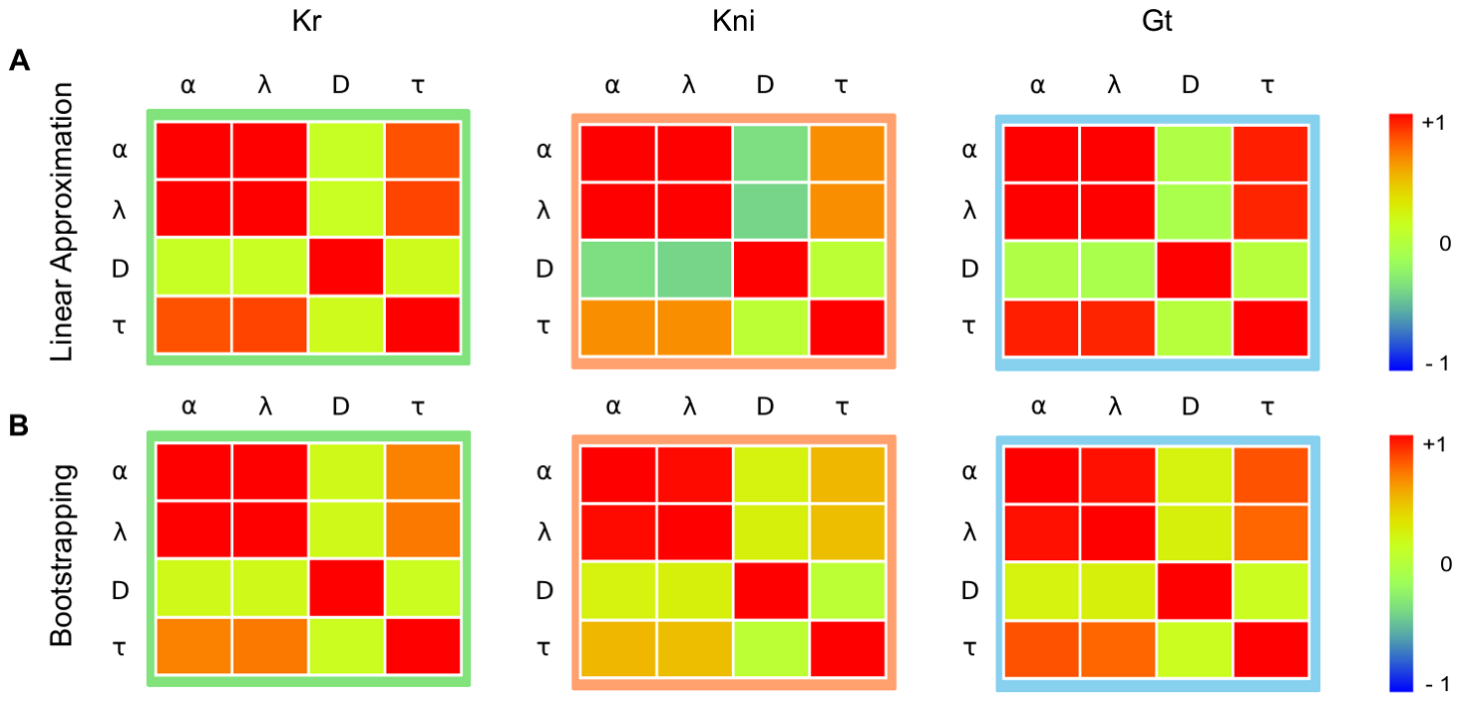
Parameter correlations. This figure shows correlation matrices for parameter values derived from linear analysis (A), and bootstrapping (B), for *Kr* (green frame), *kni* (red frame), and *gt* (blue frame). Parameter notation: 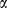 (production rate), 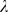 (decay rate), 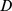 (diffusion rate), and 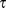 (production delay; see equation 1). Colors indicate sign and strength of correlations. Matrices in (A) are calculated from equation 6 (see Materials and Methods). Matrices in (B) are derived from the singular value decomposition of bootstrap distributions.

Correlation coefficients between parameters can be calculated from the covariance matrix (Figure 5A; see equation 6 in Materials and Methods). In all three models, correlation is high between 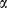 and 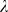. This is expected since high decay rates can compensate for high production rates. Both of these parameters are also correlated to the delays given by 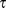. These correlations are highest for *gt*, and still very substantial for both *Kr* and *kni*. Again, this is to be expected since production delay can be mimicked to some degree by low production rates. In contrast, we found that diffusion rates are largely independent of other model parameters, except for a slight negative correlation between 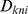 and 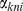, and between 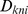 and 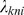. This could be due to the extremely low values of diffusion rates in all of our models, or due to the fact that diffusion affects spatial, rather than strictly local, regulatory mechanisms, which could explain the increased degree of decoupling between the two processes.

While computationally efficient, the linear identifiability analysis described above can lead to serious artifacts or biases in the estimation of confidence intervals due to its simplifying assumptions. Therefore, we validated its results by using the computationally much more expensive approach of bootstrapping [31], [71]–[73]. The bootstrap method is based on resampling protein expression patterns from distributions defined by the mean and variance of our measurements (for an equivalent analysis of the sensitivity of parameter estimates with regard to perturbations in the mRNA data see Supplementary Text S4). The model is then fitted to a large number (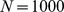 in our case) of such sampled noisy patterns. Confidence intervals and correlations for parameter estimates can be directly extracted from the resulting parameter distributions.

**Figure 6:**
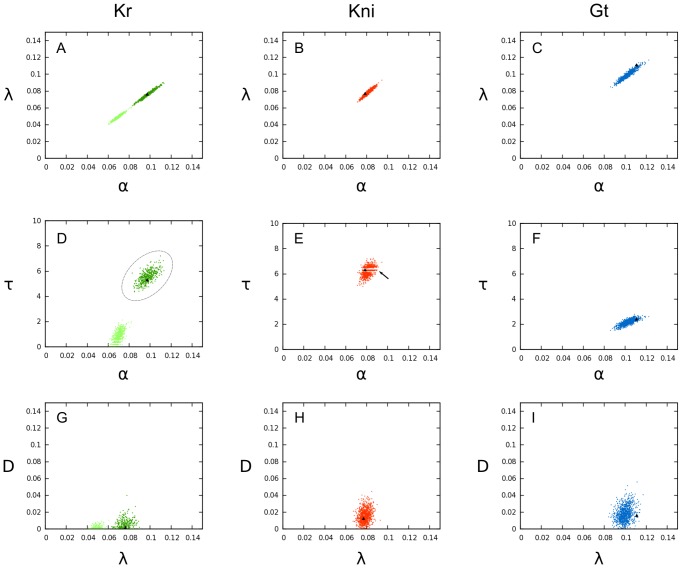
Parameter distributions obtained from bootstrapping. This figure shows illustrative examples of scatter plots for parameter values derived from 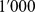 fits to simulated noisy data (sampled from the distributions of protein data measurements; see Figure 3 for mean and standard deviations of spatial expression profiles). Parameter values for *Kr* are shown in green (left column, A, D, G), for *kni* in red (centre column, B, E, H), and for *gt* in blue (right column, C, F, I). Parameter notation: 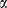 (production rate), 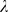 (decay rate), 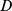 (diffusion rate), and 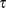 (production delay; see equation 1). Black triangles indicate the original parameter estimate obtained with unperturbed data. Dashed ellipse around parameter values for *Kr* (in D) indicates parameters selected for further analysis. Arrow in E indicates striped interference pattern in the distribution of *kni* parameter values. See text for details.

Distributions of parameter estimates obtained by bootstrapping are shown in Figure 6. In all cases, estimated parameter values are confined to relatively small subregions of search space. Only diffusion rates 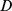 show a tendency towards saturation at their lower limit (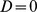; Figure 6G–I). Distributions are generally unimodal, with the exception of *Kr* which shows two distinct clusters in parameter space. The cause of this bimodal distribution remains unclear. One of the clusters (568 solutions) has implausibly small values for 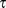 (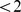 min). Therefore, we only considered the remaining 432 solutions (see dashed circle in Figure 6D) for further analysis. Another interesting feature of these parameter distributions are the two horizontal lines visible in Figure 6E, which indicate an exclusion of 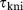 values around 6.25 and 6.30 minutes. This corresponds roughly to the time between data points, which seems to indicate that the structure of the data used for model fitting has a non-negligible impact on parameter estimation in this case.

None of these irregularities observed in parameter distributions seriously affects our ability to compute confidence intervals. This was done by determining the 95-percentile range for each parameter separately. The resulting confidence intervals and the initial guess are shown in Figure 4. The optimal solution of the unperturbed data set in every case lies within or very close (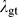) to the limits of the corresponding confidence interval (diamonds in Figure 4). With the exception of confidence intervals around 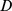, the size of bootstrap intervals lies between those of the dependent and independent intervals calculated by linear approximation. In general, this confirms the accuracy and reliability of this method.

Some notable exceptions apply. First, most confidence intervals based on bootstrapping are clearly asymmetric around the estimated optimal values. This asymmetry reflects a non-linear dependence of the optimisation problem on parameter values, which cannot be captured by confidence intervals calculated from linear approximation. Second, size of confidence intervals for 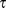 obtained by the bootstrap are frequently more similar to the size given by the dependent confidence interval, indicating that delay ranges are more accurately determinable than estimated by linear approximation.

Correlation matrices calculated by linear approximation or bootstrapping are very consistent. The anisotropic shape of parameter distributions resulting from bootstrapping reveal strong positive correlations between rate parameters for production and decay (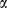 and 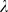; Figure 5B, 6A–C). Somewhat weaker, but nevertheless strong correlations occur between these same rate parameters and the production delays 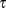 (Figure 5B, 6D–F). In contrast, diffusion rates 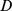 are much less correlated with any of the other parameters (Figure 5B, 6G–I).

## Discussion

In this study, we have used a reverse-engineering approach to test whether post-transcriptional regulation is required for the correct expression of gap protein domains. For this purpose, we have created a high-resolution quantitative dataset of mRNA expression patterns for the gap genes *Kr*, *kni*, and *gt* covering the entire blastoderm stage. Comparison of gap mRNA and protein expression data indicates that both are remarkably similar, although features in the mRNA data emerge a few minutes earlier than those of the corresponding protein patterns. Results of our model fits confirm this general impression: the timing and position of gap protein domains can be explained largely by a simple linear delay model, which assumes that protein patterns correspond to mRNA patterns a given amount of time in the past (plus a small contribution of protein diffusion). Based on this, we conclude that post-transcriptional regulation is not essential for gap gene mediated pattern formation. This result confirms a widely held assumption by the *Drosophila* research community that had never been put to a rigorous test.

On the other hand, our results reveal surprising and significant differences between mRNA and protein levels. In particular, our models fail to correctly reproduce both early dynamics of expression initiation (for Kni and Gt), and late maintenance of protein levels (for all three gap proteins; Figure 3; see also [58]). This indicates that temporally and spatially specific post-transcriptional regulation is required to explain these particular expression features. Only a detailed quantitative study, such as the one presented here, is able to detect such subtle nuances. No experimental evidence is currently available on the regulatory mechanisms or the functional importance of these newly discovered expression features.

Our models yield predictions that are informative and specific enough to enable focused molecular and biochemical investigations of these phenomena. The early boost in build up of Kni and Gt protein could be explained by a modulation of protein production rates. One potential mechanism for this would be a translation rate that depends on the diffusion limited arrival of mRNA molecules at the ribosomes. The maintenance of high protein levels despite rapid mRNA decay towards the end of the blastoderm stage indicates some mode of protein stability regulation. Such temporal regulation has been observed for Bcd protein [74], [75]. It could be achieved by non-linear dependence of the decay rate on protein concentration. Co-operative stability—increased longevity of dimers compared to monomers—has been proposed as a potential mechanism for increased protein half life at high concentration levels [76].

One last aspect of post-transcriptional regulation that requires our attention is the protein production delay predicted by our models. These delays, between 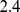 and 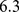 min long (Table 1), are short but yet significant enough to affect the dynamic regulatory properties of the system. They have several effects: First of all, production delays must be kept rather short to allow pattern formation on a time scale of less than 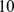 min in a system of rapidly dividing nuclei [58], [59]. There is some experimental evidence to show that this is achieved through a compact gene structure—short open reading frames with a very limited number of short introns [77]. While *kni* has a primary transcript about 3 kilobases (kb) long, its paralogue *knirps-related (knrl)* (encoding a functionally equivalent protein) contains a long intron which results in a primary RNA of about 23 kb. Its limited length allows *kni* to become expressed early, at cleavage cycle 13. In contrast, cytoplasmic mRNA of *knrl* only appears around mid cleavage cycle 14A, about half an hour later. The second aspect of the production delay is important in the context of the transient nature of gap gene patterning. While it has been shown that mRNA and protein levels of a gene converge at steady state [78], they can be significantly different when a system is far from asymptotic behavior. In the case of the gap gene system, this is reflected by the systematic anterior displacement of mRNA compared to protein domains, caused by the dynamic anterior shift of gap gene expression domains over time [22], [25], [59]. This phenomenon had been attributed to post-transcriptional regulation by some authors [67], but can now be fully explained by a combination of the anterior movement of the domains, the production delay, and the slightly different half lives of mRNA and protein.

Finally, production delays that are on the same order of magnitude as the time scale of pattern formation can lead to severe alterations of the transient dynamic behavior of the system. For example, delays can greatly increase the time it takes for the system to reach its steady stage [79], [80]. This may be functionally important for gap gene patterning, where the expression domains in the posterior half of the embryo have to be kept moving anteriorly until the onset of gastrulation (and the subsequent disappearance of gap expression), while gap domains in the central part of the embryo remain stable and reach their steady states much earlier [25], [59].

As in the case of delays, our models yield predictions of rate parameter values that are plausible, informative, and experimentally testable. Predicted decay rates imply gap protein half lives that lie between 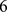 and 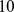 min, which is somewhat lower than the 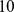 to 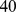 min measured for the Bcd protein [74], [75]. Our predictions of diffusion and production rates are harder to assess. The reason for this is that they are formulated in relative units, since our measurements of mRNA and protein concentrations are relative and do not yield absolute concentrations. This limitation could be overcome by emerging experimental techniques that allow the estimation of absolute levels of mRNA and protein in vivo [61], [81]–[83]. However, we can already draw some conclusions from our estimated relative values. In particular, our results indicate that gap protein diffusion must be severely restricted. This is corroborated by our observation that gap protein domains are generally only about 1–2 nuclei wider than their corresponding mRNA domains (see Supplementary Table S1), and is consistent with the model-based prediction that diffusion is not required for correct gap protein mediated patterning [22], [25]. Finally, production rates are the most difficult to measure. In this regard, the prediction of our models that protein production and decay must be quite tightly balanced may be helpful to overcome this technical limitation.

Our simple model of transcriptional regulation is limited in several important ways. We have explicitly refrained from implementing particular post-transcriptional regulatory mechanisms due to the absence of specific experimental evidence at this point. Our main aim in this current study was to first establish whether any post-transcriptional regulation is necessary for gap gene regulation. Our results clearly show that such regulation is required for the proper level, but not timing and position, of gap gene expression. Future investigations will combine experimental and data-driven modeling approaches to extend the model, and render it more mechanistically accurate.

Another limitation concerns the coarse-grained nature of our production delay. It summarizes contributions by transcriptional elongation, mRNA processing and splicing, nuclear export, and translation (see Introduction). Such coarse-graining is warranted in the light of our experimental protocols, which cannot yet distinguish between nascent and primary transcripts, or mature mRNA. A more accurate measurement of mRNA production could be achieved by using intronic probes [84]. However, this is challenging in our context due to the extremely compact structure of gap genes. Another way to address this issue would be to formulate a model with a distributed production delay, reflecting the fact that the measured mRNAs are at different stages of their maturation process. We have not implemented such distributed effects in our current model since the benefit in terms of biological insight would be limited, while estimating a distribution of parameter values would pose significant technical challenges for model fitting.

The last, and most important, limitation of our current approach is that transcriptional and post-transcriptional regulatory processes involved in gap gene patterning are still implemented in different models. It is our aim to synthesise both of these stages into a regulatory network model featuring realistic production delays. We expect that such a model would solve several important issues. For instance, current gene network models still fail to reproduce the early regulatory and expression dynamics—based on regulatory inputs from maternal gradients only—in an accurate and biologically plausible manner [22], [36], [58]. An integrated model featuring a true expression delay will undoubtedly help to overcome this problem. Both the quantitative dataset of mRNA expression, and the mathematical analysis of gap gene translation presented here will be crucial for the implementation of such a model.

At a more general scale, we have provided a proof of principle that rigorous model fitting and parameter identifiability analysis are possible in the context of the complex regulation of animal development. We hope that this will enable a more widespread and rigorous application of reverse-engineering approaches to problems of biological pattern formation. In our view, this constitutes an important methodological advance, which is crucial to apply the considerable potential of quantitative reverse-engineering strategies for our understanding of development.

## Materials and Methods

### Data Acquisition

Blastoderm stage embryos of *Drosophila melanogaster* (raised at 25°C) were collected 1–4 hrs after egg laying. Embryos were fixed and stained using FITC- (*Kr*, *gt*) or DIG-labeled (*kni*) riboprobes, plus polyclonal antiserum against Even-Skipped (Eve) [85], according to standard experimental protocols [58], [86]–[88]. Nuclei were counterstained using Hoechst 34580. Imaging took place on a Leica TCS SP5 confocal microscope using a 20× objective, and an additional digital zoom of 1.3x. We imaged the blastodermal nuclear layer of laterally oriented embryos at two 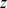-positions, 1.0–1.2 μm apart. Data channels were scanned sequentially at a resolution of 1024×1024 pixels. Only embryos at cleavage cycle 14A (C14A) [89] were chosen for further processing. For earlier time points, we use previously published gap mRNA expression data [58].

### Data Processing

Data processing and quantification methods are described elsewhere in detail [58], [69]. In brief, we create a binary whole-embryo mask by thresholding, which is used to automatically crop and align embryo images such that anterior is left, dorsal up. We identify nuclei and their surrounding territories of cytoplasm using watershed-based image segmentation algorithms [58], [57]. From these watershed masks, we determine the position of nuclei, as well as the concentration of mRNA (in nuclei plus surrounding cytoplasm), and Eve protein (in nuclei only). Embryos are classified into eight time classes (T1–8; each 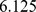 min long) during C14A, based on Eve expression patterns and morphological markers [69]. Non-specific background staining is removed as previously described [90]. Expression data are registered using a spline-based approach [91]. Background removal and data registration are implemented in an integrated tool [92]. Finally, registered mRNA data within a lateral strip (covering 10% of the embryo) are placed into 100 bins along the A–P axis, and concentration values in each bin are averaged per gene and time class. Individual expression profiles, integrated patterns, and the number of embryos used for each time class, are shown in Supplementary Figures S1, S2, S3. Gap mRNA expression patterns for C10–C13 were taken from [58], and scaled to provide a smooth transition between the two datasets. Integrated protein expression data used in our analysis are from the FlyEx database: http://urchin.spbcas.ru/flyex [57], [60]. We normalise our mRNA data (using the same scaling factor for all time classes) by adjusting peak concentrations to the maximum expression level observed for protein. Expression peaks, domain boundary positions (points of 50% maximum expression), and domain widths were calculated from spline approximations to the expression data as described in [63].

### Model Structure and Numerical Solver

The basic objects of our model represent nuclei plus their associated surrounding cytoplasm (energids). The state variables of the model describe the concentration of intra-nuclear gap protein within each energid. Change in gap protein concentration across time and space is described by a system of ordinary differential equations (ODEs; see equation 1 in the Introduction), and depends on protein production from mRNA (concentration averaged across both nuclear and cytoplasmic portions of the energid), protein diffusion between energids, and protein decay. The model spans the entire blastoderm stage, from 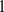 min after the onset of cleavage cycle 10 (C10; 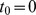 min) to the end of C14A at the onset of gastrulation (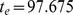 min) [89]. During this time, four mitotic divisions occur (division 10, 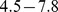 min; division 11, 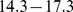 min; division 12, 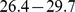 min; division 13, 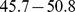 min; [89]). During mitosis, transcription of mRNA is interrupted and unfinished transcripts are actively degraded [93]. This process has never been quantified. Here, we assume fast mitotic mRNA degradation: therefore, the protein production term in our model (see equation 1) is set to zero, whenever the time point 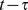 (current time minus the production delay) comes to lie within the time of mitosis. At the end of each mitotic phase, nuclei divide instantaneously (and thus the number of ODEs in the model increases approximately two-fold), and the distance between them is halved. Due to the presence of diffusion, our ODEs are coupled across space. The spatial range of our model is defined for each gap gene independently. In general, models cover most of the segmented trunk region of the embryo. Ranges were defined to include the posterior boundary of the anterior Gt domain, the central Kr domain, the abdominal Kni domain, and the posterior domain of Gt (*Kr*: 25.5–88.5%, *kni*: 32.5–88.5%, *gt*: 32.5–95.5% A–P position, where 0% is the anterior pole).

Although our models are feed-forward and linear, they depend on a non-linear external input (the mRNA expression profiles). Therefore, we solve these systems of ODEs numerically using an implementation of the MATLAB *dde23* solver in C [94]. The solver was modified to satisfy the following requirements: (i) it must be able to provide dense output at time points for which there is no external input data; (ii) it must be able to handle discontinuities propagating through the system due to the delay history; and (iii) it has to handle implicit formulas if the stepsize becomes bigger than the delay. We use linear interpolation between data points to provide mRNA concentrations as external inputs at arbitrary points.

### Structural Identifiability Analysis

Structural parameter identifiability analysis was performed using the Laplace transform based approach [28]. The idea is to obtain the transfer matrix of the system in rational canonical form and to assess whether the transfer matrix is unique. Supplementary Text S2 provides a detailed description of this approach, and the calculations we performed.

### Parameter Estimation

Numerical solutions are produced for time points C10–C13, and T1–T8 within cleavage cycle 14A. We then calculate a weighted sum of squared differences (according to equation 2), which is minimised using two alternative optimisation strategies. The first of these consists of global optimisation using (parallel) Lam Simulated Annealing (pLSA; [95]–[98]). This method is reliable and robust, and has been successfully used in previous reverse-engineering studies of the gap gene system [22], [58], [64]. pLSA is computationally intensive and was implemented in C. The second approach consists of a scatter search approach (eSS), which systematically explores parameter space, triggering a local gradient-based search [99] whenever a promising parameter set has been found [100]–[104]. eSS is implemented in the AMIGO toolbox (based on MATLAB; [105]). Both strategies resulted in virtually identical model fits and parameter estimates (Figure 3; Table 1).

The following search space limits were used for optimisation. 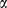: 0.005–5.0; 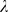: 0.0347–0.6931; 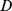: 0.0–0.3; 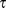: 0.0–10.0.

The quality of the fit between data and model output is measured by the root mean square (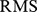) score, which represents the average difference between modeled and measured protein concentrations across all data points:
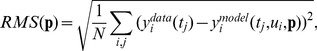
(3)where 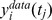 denotes the concentrations of protein 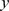 in nucleus 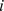 at timepoint 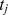, while 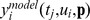 corresponds to the according simulated value of protein concentration dependent on the chosen parameter set 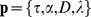. The RMS—unlike the weighted least square sum (see equation (2))—is independent of not only the noise in measurement but also the number of data points used for fitting. It therefore makes model fits of different gap genes comparable to each other on a quantitative basis.

### Practical Identifiability Analysis

We used two alternative strategies for practical parameter identifiability analysis. The first one is based on a local linear approximation of the ‘energy’-landscape given by the objective function (2) around a given optimum as described in [35], [36], [66]. Estimates of the local contour of this landscape are used to determine an ellipsoidal confidence region around the optimal parameter set obtained by eSS. Dependent confidence intervals (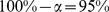) are then given by the intersection of the ellipsoid with the parameter axes
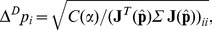
(4)while independent confidence intervals are specified by the projection of the ellipsoid onto the parameter axes
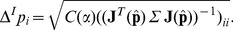
(5)


Correlations among model parameters can be calculated based on the covariance matrix 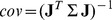 as
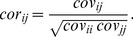
(6)In all equations, diagonal entries in 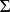 correspond to 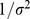 with 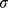 beeing the standard deviation on measurement. 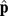 is the inferred parameter set obtained via eSS and 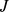 denotes the sensitivity matrix of the model given by the first order derivative of the observables with respect to the parameters. 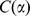 is calculated as the upper 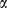 part of Fishers distribution with 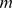 and 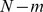 degrees of freedom
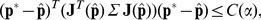
(7)with
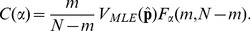
(8)


The second strategy is based on a bootstrapping approach, where we sample a normal distribution (based on measured means and variances) for each of our protein expression data points. Data points for which no variance estimates were available were not randomised. Resulting sampled expression profiles were corrected by setting negative concentration values to zero. 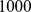 bootstrapping samples were generated for each gap gene in this way. These samples were then fitted by pLSA as described above. Bootstrapping runs were performed in parallel on a cluster provided by the Spanish Supercomputing Network (RES—Red Española de Supercomputación). From the resulting distribution of parameter values, we directly calculate 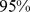 confidence intervals, plus correlation coefficients indicating mutual dependence of model parameters. In the case of *Kr*, which shows a bimodal parameter distribution (Figure 6), we only considered a subset of the sampled estimates (see Results). For an analysis of the sensitivity of parameter values to changes in mRNA expression patterns, analogous to the protein bootstrap, please refer to Supplementary Text S4.

## Supporting Information

Figure S1 **Quantification of** ***Kr*** **mRNA data.** Each panel represents a time class (T1–T8) in C14A showing an example embryo image (top), un-registered expression profiles (middle), and integrated expression patterns (bottom, with standard deviations shown as dark grey background). Embryo images show lateral views: anterior is to the left, dorsal up. Graphs plot relative mRNA concentration against A–P Position (in %, where 0% is the anterior pole). Expression profiles consider only the central 10% strip along the dorso-ventral axis. Green profiles in middle panels were extracted from embryos shown in images above. Lightly shaded background in lower panels represents the region of the embryo considered in our models. See Materials and Methods for details on data processing.
(PDF)

Figure S2 **Quantification of** ***kni*** **mRNA data.** Each panel represents a time class (T1–T8) in C14A showing an example embryo image (top), un-registered expression profiles (middle), and integrated expression patterns (bottom, with standard deviations shown as dark grey background). Embryo images show lateral views: anterior is to the left, dorsal up. Graphs plot relative mRNA concentration against A–P Position (in %, where 0% is the anterior pole). Expression profiles consider only the central 10% strip along the dorso-ventral axis. Red profiles in middle panels were extracted from embryos shown in images above. Lightly shaded background in lower panels represents the region of the embryo considered in our models. See Materials and Methods for details on data processing.
(PDF)

Figure S3 **Quantification of** ***gt*** **mRNA data.** Each panel represents a time class (T1–T8) in C14A showing an example embryo image (top), un-registered expression profiles (middle), and integrated expression patterns (bottom, with standard deviations shown as dark grey background). Embryo images show lateral views: anterior is to the left, dorsal up. Graphs plot relative mRNA concentration against A–P Position (in %, where 0% is the anterior pole). Expression profiles consider only the central 10% strip along the dorso-ventral axis. Blue profiles in middle panels were extracted from embryos shown in images above. Lightly shaded background in lower panels represents the region of the embryo considered in our models. See Materials and Methods for details on data processing.
(PDF)

Figure S4 **Positional variability in gap domain features.** This figures shows standard deviations for the position of characteristic features of the central *Kr* domain (left), the abdominal *kni* domain (center), and the posterior *gt* domain (right; see also Supplementary Table S1). Data for mRNA shown as solid lines, for protein as dashed lines. ‘Maximum’ corresponds to the domain peak or maximum; ‘Anterior’ is the position of the anterior boundary, ‘Posterior’ that of the posterior boundary (determined as the position of 50% maximum concentration levels in each domain); ‘Domain width’ corresponds to the distance between anterior and posterior boundaries. Positions are indicated in % A–P embryo length. We only plot time points T1–T6, as low mRNA expression levels at T7/T8 make a precise quantification of variability impossible at those stages.
(PDF)

Figure S5
**Parameter distributions of 100 pLSA optimisation runs.** This Figure shows illustrative examples of scatter plots for parameter values derived from 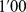 fits. Parameter values for *Kr* are shown in green (left column, A, D, G), for *kni* in red (center column, B, E, H), and for *gt* in blue (right column, C, F, I). Parameter notation: 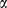 (production rate), 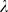 (decay rate), 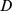 (diffusion rate), and 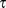 (production delay; see equation 1 of the main text). Since we applied the stochastic optimisation method pLSA, we checked whether parameter estimates of multiple (100) optimisation runs would show any significant deviation from one another. However, parameter estimates turned out to be tightly confined in parameter space, supporting evidence that pLSA robustly recovers the same solution across runs. Limits for axes are chosen according to Figure 6 of the main paper.(PDF)

Figure S6
**Parameter estimation with fixed delays.** In order to test whether we can determine the value of delay parameters 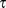 correctly, we performed a series of runs for the *gt* model, fixing 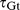 to values between 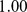 and 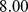 min (with a step size of 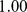 min between series of optimisation runs). Resulting WLS scores 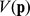 are shown as black dots. For comparison, the red triangle indicates the WLS score of the model obtained by estimating 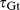. The inset shows a detailed view of the interval between 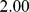 and 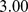 which we sampled more intensively, with a step size of 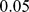. Optimal parameter values (for red-triangle solutions) are indicated on the right.(PDF)

Table S1
**Comparison of domain position and width between mRNA and protein data.** Mean (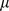) and variances (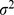) of the position of expression peaks (‘max’), domain boundary positions (‘A’, anterior; ‘P’, posterior), and domain widths are shown for the central domain of *Kr* (green), the abdominal domain of *kni* (red), and the posterior domain of *gt* (blue).(PDF)

Text S1 **Testing significance of mRNA decay during late C14 using the two-sided Kolmogorow-Smirnow-Test.**
(PDF)

Text S2 **Structural identifiability analysis.**
(PDF)

Text S3 **Comparison of global optimization solvers.**
(PDF)

Text S4 **Sensitivity of parameter estimates to mRNA data.**
(PDF)
