# Supplementary material for: Reverse-Engineering Post-Transcriptional Regulation of Gap Genes in Drosophila melanogaster
Source: PLoS Comput Biol. 2013 Oct 31;9(10):e1003281. doi: 10.1371/journal.pcbi.1003281 (PMC3814631; doi:10.1371/journal.pcbi.1003281)
Supplement: Figure S1 — Quantification of Kr mRNA data. Each panel represents a time class (T1–T8) in C14A showing an example embryo image (top), un-registered expression profiles (middle), and integrated expression patterns (bottom, with standard deviations shown as dark grey background). Embryo images show lateral views: anterior is to the left, dorsal up. Graphs plot relative mRNA concentration against A–P Position (in %, where 0% is the anterior pole). Expression profiles consider only the central 10% strip along the dorso-ventral axis. Green profiles in middle panels were extracted from embryos shown in images above. Lightly shaded background in lower panels represents the region of the embryo considered in our models. See Materials and Methods for details on data processing. (PDF) [file pcbi.1003281.s001.pdf]

Supplementary Figure S1: *Kr* mRNA expression data.

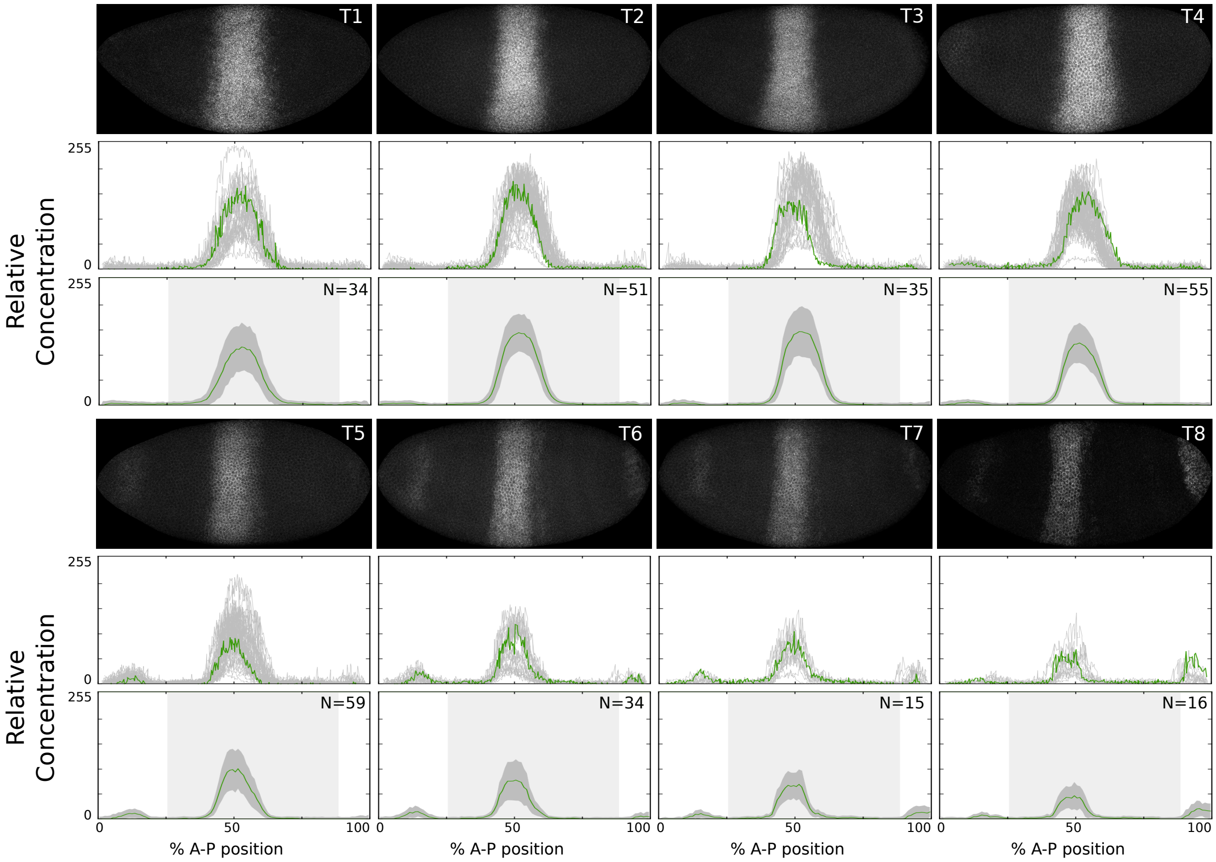

Figure S1. Quantification of *Kr* mRNA Data. Each panel represents a time class (T1–T8) in C14A showing an example embryo image (top), un-registered expression profiles (middle), and integrated expression patterns (bottom, with standard deviations shown as dark grey background). Embryo images show lateral views: anterior is to the left, dorsal up. Graphs plot relative mRNA concentration against A–P Position (in %, where 0% is the anterior pole). Expression profiles consider only the central 10% strip along the dorso-ventral axis. Green profiles in middle panels were extracted from embryos shown in images above. Lightly shaded background in lower panels represents the region of the embryo considered in our models. See Materials and Methods for details on data processing.
