# Supplementary material for: Reverse-Engineering Post-Transcriptional Regulation of Gap Genes in Drosophila melanogaster
Source: PLoS Comput Biol. 2013 Oct 31;9(10):e1003281. doi: 10.1371/journal.pcbi.1003281 (PMC3814631; doi:10.1371/journal.pcbi.1003281)
Supplement: Figure S4 — Positional variability in gap domain features. This figures shows standard deviations for the position of characteristic features of the central Kr domain (left), the abdominal kni domain (center), and the posterior gt domain (right; see also Supplementary Table S1). Data for mRNA shown as solid lines, for protein as dashed lines. ‘Maximum’ corresponds to the domain peak or maximum; ‘Anterior’ is the position of the anterior boundary, ‘Posterior’ that of the posterior boundary (determined as the position of 50% maximum concentration levels in each domain); ‘Domain width’ corresponds to the distance between anterior and posterior boundaries. Positions are indicated in % A–P embryo length. We only plot time points T1–T6, as low mRNA expression levels at T7/T8 make a precise quantification of variability impossible at those stages. (PDF) [file pcbi.1003281.s004.pdf]

Supplementary Figure S4: Positional variability in gap domain features.

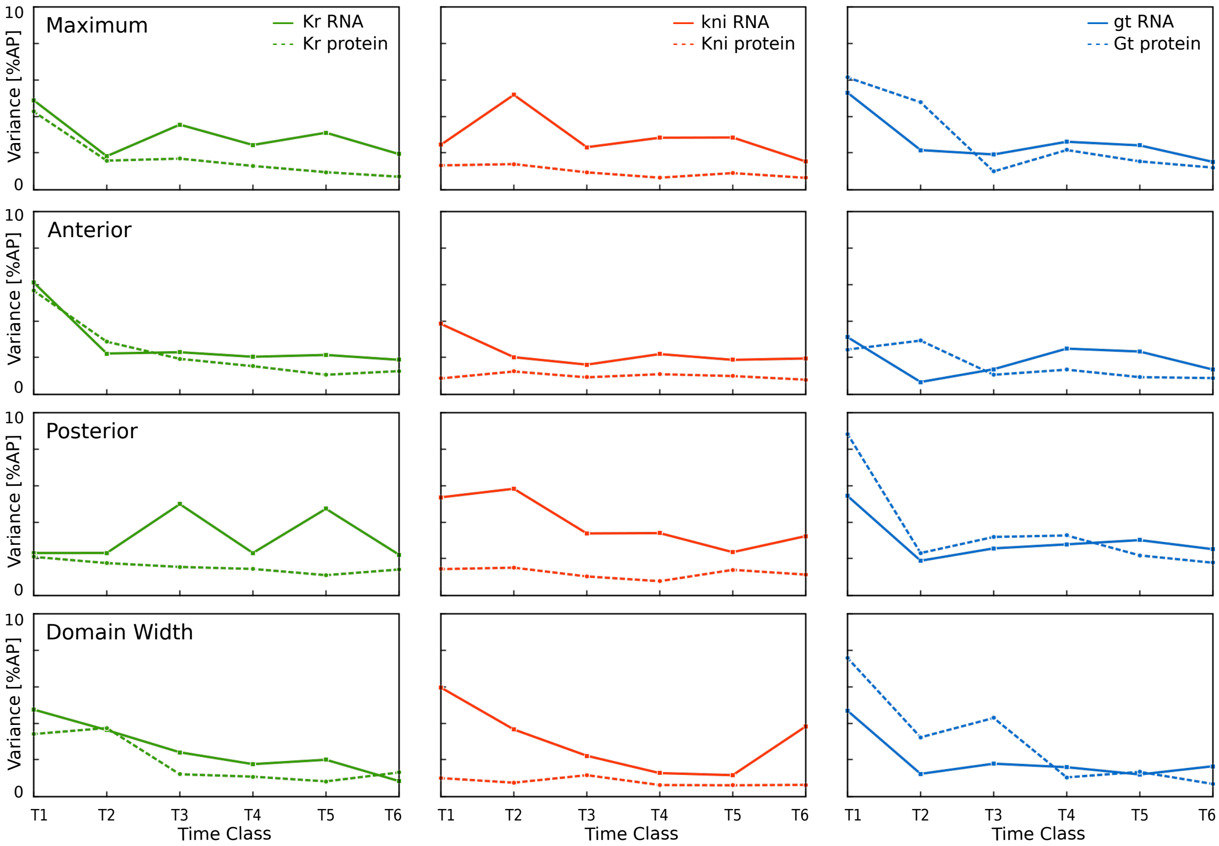

Figure S4. Positional variability in gap domain features. This figures shows standard deviations for the position of characteristic features of the central *Kr* domain (left), the abdominal *kni* domain (center), and the posterior *gt* domain (right; see also Supplementary Table S1). Data for mRNA shown as solid lines, for protein as dashed lines. 'Maximum' corresponds to the domain peak or maximum; 'Anterior' is the position of the anterior boundary, 'Posterior' that of the posterior boundary (determined as the position of 50% maximum concentration levels in each domain); 'Domain width' corresponds to the distance between anterior and posterior boundaries. Positions are indicated in % A–P embryo length. We only plot time points T1–T6, as low mRNA expression levels at T7/T8 make a precise quantification of variability impossible at those stages.
