# Supplementary material for: Reverse-Engineering Post-Transcriptional Regulation of Gap Genes in Drosophila melanogaster
Source: PLoS Comput Biol. 2013 Oct 31;9(10):e1003281. doi: 10.1371/journal.pcbi.1003281 (PMC3814631; doi:10.1371/journal.pcbi.1003281)
Supplement: Figure S5 — Parameter distributions of 100 pLSA optimisation runs. This Figure shows illustrative examples of scatter plots for parameter values derived from fits. Parameter values for Kr are shown in green (left column, A, D, G), for kni in red (center column, B, E, H), and for gt in blue (right column, C, F, I). Parameter notation: (production rate), (decay rate), (diffusion rate), and (production delay; see equation 1 of the main text). Since we applied the stochastic optimisation method pLSA, we checked whether parameter estimates of multiple (100) optimisation runs would show any significant deviation from one another. However, parameter estimates turned out to be tightly confined in parameter space, supporting evidence that pLSA robustly recovers the same solution across runs. Limits for axes are chosen according to Figure 6 of the main paper. (PDF) [file pcbi.1003281.s005.pdf]

Supplementary Figure S5: Parameter distributions of multiple pLSA optimisation runs.

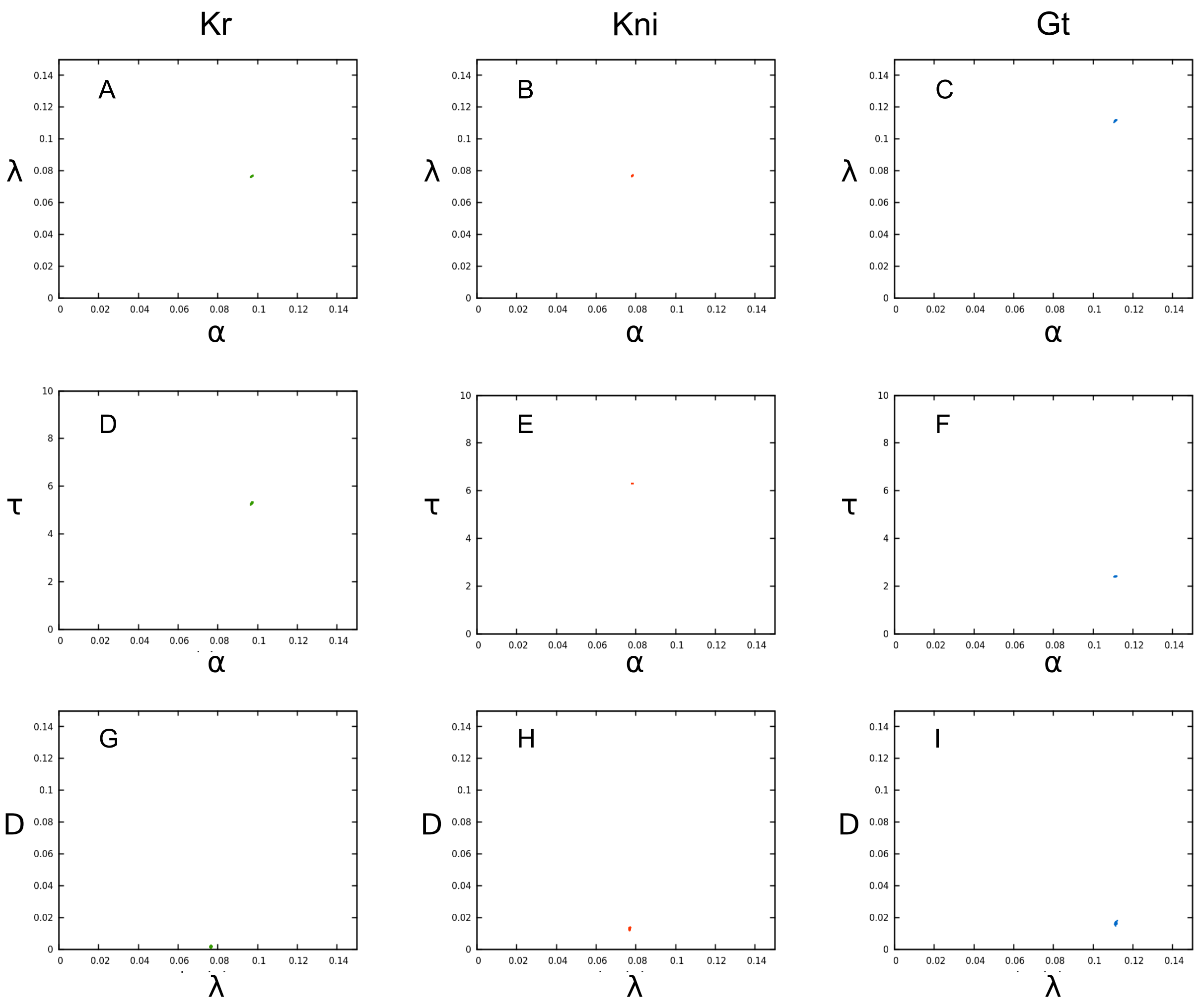

Figure S5. Parameter distributions of 100 pLSA optimisation runs.
This Figure shows illustrative examples of scatter plots for parameter values derived from 1′00 fits. Parameter values for *Kr* are shown in green (left column, A, D, G), for *kni* in red (center column, B, E, H), and for *gt* in blue (right column, C, F, I). Parameter notation: α (production rate), λ (decay rate), D (diffusion rate), and τ (production delay; see equation 1 of the main text). Since we applied the stochastic optimisation method pLSA, we checked whether parameter estimates of multiple (100) optimisation runs would show any significant deviation from one another. However, parameter estimates turned out to be tightly confined in parameter space, supporting evidence that pLSA robustly recovers the same solution across runs. Limits for axes are chosen according to Figure 6 of the main paper.
