# Supplementary material for: Reverse-Engineering Post-Transcriptional Regulation of Gap Genes in Drosophila melanogaster
Source: PLoS Comput Biol. 2013 Oct 31;9(10):e1003281. doi: 10.1371/journal.pcbi.1003281 (PMC3814631; doi:10.1371/journal.pcbi.1003281)
Supplement: Figure S6 — Parameter estimation with fixed delays. In order to test whether we can determine the value of delay parameters correctly, we performed a series of runs for the gt model, fixing to values between and min (with a step size of min between series of optimisation runs). Resulting WLS scores are shown as black dots. For comparison, the red triangle indicates the WLS score of the model obtained by estimating . The inset shows a detailed view of the interval between and which we sampled more intensively, with a step size of . Optimal parameter values (for red-triangle solutions) are indicated on the right. (PDF) [file pcbi.1003281.s006.pdf]

Supplementary Figure S6: Parameter estimation with fixed delays.

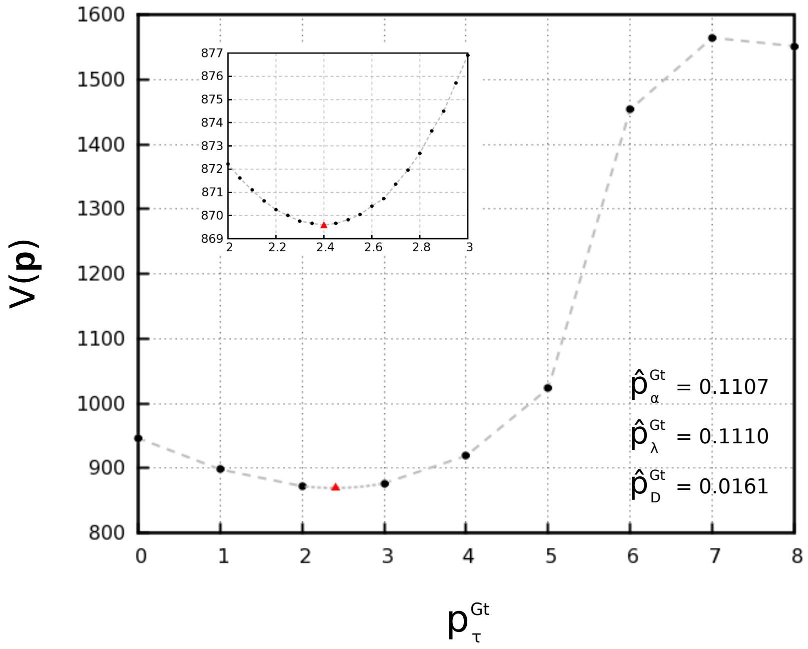

Figure S6. Parameter estimation with fixed delays. In order to test whether we can determine the value of delay parameters τ correctly, we performed a series of runs for the *gt* model, fixing τ_{Gt} to values between 1.00 and 8.00 min (with a step size of 1.00 min between series of optimisation runs). Resulting WLS scores $V(\mathbf{p})$ are shown as black dots. For comparison, the red triangle indicates the WLS score of the model obtained by estimating τ_{Gt}. The inset shows a detailed view of the interval between 2.00 and 3.00 which we sampled more intensively, with a step size of 0.05. Optimal parameter values (for red-triangle solutions) are indicated on the right.
