# Supplementary material for: Reverse-Engineering Post-Transcriptional Regulation of Gap Genes in Drosophila melanogaster
Source: PLoS Comput Biol. 2013 Oct 31;9(10):e1003281. doi: 10.1371/journal.pcbi.1003281 (PMC3814631; doi:10.1371/journal.pcbi.1003281)
Supplement: Table S1 — Comparison of domain position and width between mRNA and protein data. Mean () and variances () of the position of expression peaks (‘max’), domain boundary positions (‘A’, anterior; ‘P’, posterior), and domain widths are shown for the central domain of Kr (green), the abdominal domain of kni (red), and the posterior domain of gt (blue). (PDF) [file pcbi.1003281.s007.pdf]

Supplementary Table S1: Comparison of domain position and width.

	Time	$\mu(x_{max})$	$\sigma^2(x_{max})$	$\mu(x_A)$	$\sigma^2(x_A)$	$\mu(x_P)$	$\sigma^2(x_P)$	$\mu(x_{width})$	$\sigma^2(x_{width})$
Kr mRNA	T1	52.57	4.88	44.95	6.10	60.22	2.31	15.27	4.75
	T2	52.49	1.82	45.03	2.22	60.33	2.31	15.30	3.63
	T3	52.26	3.55	45.06	2.29	60.30	4.99	15.24	2.41
	T4	51.55	2.44	45.00	2.04	59.06	2.31	14.07	1.76
	T5	50.18	3.11	44.39	2.14	56.78	4.73	12.39	2.01
	T6	49.44	1.94	43.83	1.88	55.16	2.22	11.32	0.84
	T7	48.54	2.10	42.65	1.59	54.07	2.86	11.42	1.91
	T8	48.20	3.89	42.90	5.03	53.46	3.45	10.56	1.16

	Time	$\mu(x_{max})$	$\sigma^2(x_{max})$	$\mu(x_A)$	$\sigma^2(x_A)$	$\mu(x_P)$	$\sigma^2(x_P)$	$\mu(x_{width})$	$\sigma^2(x_{width})$
Kr Protein	T1	53.50	4.26	44.33	5.66	62.40	2.10	18.08	3.41
	T2	52.51	1.58	43.84	2.87	61.30	1.76	17.46	3.74
	T3	52.15	1.69	44.39	1.93	60.16	1.55	15.77	1.21
	T4	52.13	1.29	44.22	1.53	60.11	1.44	15.89	1.08
	T5	51.62	0.95	43.91	1.06	59.56	1.10	15.65	0.82
	T6	51.15	0.70	43.61	1.26	59.19	1.41	15.57	1.31
	T7	50.18	1.49	43.22	1.45	57.87	2.04	14.64	2.01
	T8	49.89	0.90	43.28	1.19	57.16	2.01	13.88	1.66

	Time	$\mu(x_{max})$	$\sigma^2(x_{max})$	$\mu(x_A)$	$\sigma^2(x_A)$	$\mu(x_P)$	$\sigma^2(x_P)$	$\mu(x_{width})$	$\sigma^2(x_{width})$
kni mRNA	T1	65.13	2.45	59.87	3.84	70.81	5.36	10.94	5.96
	T2	64.93	5.18	59.55	2.02	70.43	5.83	10.88	3.66
	T3	64.01	2.31	58.70	1.61	69.11	3.38	10.41	2.22
	T4	63.38	2.83	58.81	2.19	67.75	3.40	8.94	1.27
	T5	61.60	2.85	57.25	1.88	65.81	2.36	8.56	1.16
	T6	60.18	1.53	55.86	1.95	64.17	3.24	8.31	3.83
	T7	61.32	5.68	56.43	6.61	65.99	35.49	9.56	33.75
	T8	60.52	4.24	53.37	55.31	65.29	0.53	11.93	58.53

	Time	$\mu(x_{max})$	$\sigma^2(x_{max})$	$\mu(x_A)$	$\sigma^2(x_A)$	$\mu(x_P)$	$\sigma^2(x_P)$	$\mu(x_{width})$	$\sigma^2(x_{width})$
Kni Protein	T1	65.79	1.32	58.81	0.86	73.97	1.43	15.16	1.00
	T2	64.66	1.39	58.44	1.24	71.22	1.51	12.78	0.75
	T3	64.80	0.94	59.01	0.92	70.68	1.03	11.67	1.16
	T4	64.03	0.65	58.82	1.09	69.82	0.77	11.00	0.62
	T5	63.81	0.90	58.39	0.99	69.38	1.39	10.98	0.61
	T6	63.57	0.64	58.39	0.79	68.99	1.13	10.61	0.63
	T7	63.06	1.09	57.85	0.79	68.34	1.08	10.50	0.26
	T8	62.60	0.84	57.14	0.60	67.65	1.03	10.52	0.69

	Time	$\mu(x_{max})$	$\sigma^2(x_{max})$	$\mu(x_A)$	$\sigma^2(x_A)$	$\mu(x_P)$	$\sigma^2(x_P)$	$\mu(x_{width})$	$\sigma^2(x_{width})$
gt mRNA	T1	75.67	5.30	68.72	3.12	82.65	5.43	13.93	4.68
	T2	74.28	2.16	68.06	0.67	79.51	1.89	11.45	1.23
	T3	72.38	1.92	67.16	1.36	77.58	2.57	10.42	1.79
	T4	72.14	2.61	67.45	2.49	77.21	2.78	9.76	1.60
	T5	70.85	2.42	66.68	2.33	75.59	3.02	8.91	1.20
	T6	69.55	1.51	65.71	1.35	73.56	2.52	7.85	1.65
	T7	68.26	1.79	64.08	1.49	71.88	1.73	7.80	1.31
	T8	68.08	1.65	63.79	1.92	71.97	2.81	8.18	4.74

	Time	$\mu(x_{max})$	$\sigma^2(x_{max})$	$\mu(x_A)$	$\sigma^2(x_A)$	$\mu(x_P)$	$\sigma^2(x_P)$	$\mu(x_{width})$	$\sigma^2(x_{width})$
Gt Protein	T1	81.00	6.13	72.67	2.44	90.12	8.82	17.55	7.57
	T2	77.78	4.77	71.01	2.93	84.76	2.31	14.00	3.23
	T3	75.00	0.99	69.33	1.06	82.26	3.19	12.92	4.29
	T4	74.14	2.16	68.67	1.34	79.77	3.27	11.09	1.04
	T5	73.00	1.54	68.01	0.93	78.48	2.18	10.47	1.34
	T6	72.22	1.20	67.32	0.88	77.51	1.78	10.19	0.68
	T7	71.35	1.32	66.82	0.89	76.36	1.15	9.54	0.23
	T8	70.66	1.35	66.29	1.16	75.48	1.50	9.19	0.30

Table S1. Comparison of domain position and width between mRNA and protein data. Mean (μ) and variances (σ^2) of the position of expression peaks ('max'), domain boundary positions ('A', anterior; 'P', posterior), and domain widths are shown for the central domain of *Kr* (green), the abdominal domain of *kni* (red), and the posterior domain of *gt* (blue).
