# Supplementary material for: Reverse-Engineering Post-Transcriptional Regulation of Gap Genes in Drosophila melanogaster
Source: PLoS Comput Biol. 2013 Oct 31;9(10):e1003281. doi: 10.1371/journal.pcbi.1003281 (PMC3814631; doi:10.1371/journal.pcbi.1003281)
Supplement: Text S1 — Testing significance of mRNA decay during late C14 using the two-sided Kolmogorow-Smirnow-Test. (PDF) [file pcbi.1003281.s008.pdf]

Supplementary Text S1: Testing significance of mRNA decay during late C14 using the two-sided Kolmogorow-Smirnow-Test.

In order to test whether the observed decrease of mRNA expression levels during late cell cycle 14A is significant, we have carried out a two–sided Kolmogorov–Smirnov test. Here, we compare mRNA expression patterns of all three gap genes between the time points of highest expression (T4–T7) and mRNA expression at the end of C14A (T8). As can be seen from Supplementary Table S1, mRNA expression patterns at T4 and T8 are significantly different for all the gap genes.

Table S2. Test Statistics of the two sided Kolmogorow-Smirnow-Test. Shown are H and p values for a two sided Kolmogorow-Smirnow-Test comparing protein and mRNA expression profiles of each time point between T4 and T8 with the corresponding expression profile at time point T8. $H = 1$ signifies, that the hypotheses of both expression patterns being drawn from the same distribution must be rejected with a significance level of 5%.

		T4	T5	T6	T7	T8
protein	Kr	*H=1,p=0.0054*	*H=0,p=0.1250*	*H=0,p=0.8164*	*H=0,p=0.2741*	*H=0,p=1*
	Kni	*H=0,p=0.8935*	*H=0,p=0.7558*	*H=0,p=0.9750*	*H=0,p=0.3139*	*H=0,p=1*
	Gt	*H=0,p=0.6701*	*H=0,p=0.9293*	*H=0,p=0.9865*	*H=0,p≈1.0000*	*H=0,p=1*

		T4	T5	T6	T7	T8
mRNA	*Kr*	*H=1,p=0.03*	*H=1,p=0.0499*	*H=0,p=0.1882*	*H=0,p=0.5198*	*H=0,p=1*
	kni	*H=1,p≈0.00*	*H=1,p≈0.00*	*H=1,p=0.0012*	*H=1,p≈0.00*	*H=0,p=1*
	gt	*H=1,p=0.0499*	*H=0,p=0.0803*	*H=0,p=0.3849*	*H=0,p=0.9293*	*H=0,p=1*
