# Supplementary material for: Reverse-Engineering Post-Transcriptional Regulation of Gap Genes in Drosophila melanogaster
Source: PLoS Comput Biol. 2013 Oct 31;9(10):e1003281. doi: 10.1371/journal.pcbi.1003281 (PMC3814631; doi:10.1371/journal.pcbi.1003281)
Supplement: Text S2 — Structural identifiability analysis. (PDF) [file pcbi.1003281.s009.pdf]

Supplementary Text S2: Structural identifiability analysis.

Structural parameter identifiability analysis is performed under the assumption of an ideal scenario where noise-free time-continuous experimental data are available. The objective is to answer the question whether under those ideal conditions the parameters can be given unique values within a given search space (see also main text). It is worth remarking at this point, that structural identifiability analysis is different to practical identifiability analysis in the sense that the actual experimental conditions and available data are not considered, only model structure and an idealised version of the experimental design is taken into account. Therefore, structural identifiability analysis answers the question whether model parameters can be determined at all, given a certain type of model and a certain type of experimental scheme (observed quantities and stimulation conditions).

The model in Eq.(1) in the main text can be rewritten in matrix form as follows:

$$M(\boldsymbol{\theta}): \qquad \dot{\mathbf{y}} = \alpha \mathbf{B}\mathbf{u}(t-\tau) + \mathbf{A}\mathbf{y} \tag{1}$$

where $\mathbf{y} \geq \mathbf{0}$, $\dot{\mathbf{y}} \in R^n$ correspond to the protein concentrations and their time derivatives; $\mathbf{B} \in R^{n\times n}$ is the control matrix, the identity matrix in this case; $\mathbf{u}(t-\tau) \geq \mathbf{0} \in R^n$ corresponds to the vector of delayed inputs and $\mathbf{A} \in R^{n\times n}$ is the so called state matrix, defined as the following tridiagonal symmetric matrix:

$$\mathbf{A} = \begin{bmatrix} a_0 & D & & & & & \\ D & a_1 & D & & & & \\ & D & a_1 & D & & & \\ & & D & a_1 & D & & \\ & & & \ddots & \ddots & \ddots & \\ & & & & D & a_1 & D \\ & & & & & D & a_0 \end{bmatrix}$$

with $a_0 = -D - \lambda$ and $a_1 = -2D - \lambda$.

Given a reference model $M(\boldsymbol{\theta}^*)$ defined as:

$$M(\boldsymbol{\theta}^*): \qquad \dot{\mathbf{y}} = \alpha^* \mathbf{B}\mathbf{u}(t-\tau^*) + \mathbf{A}^*\mathbf{y} \tag{2}$$

in which the system parameters $\boldsymbol{\theta} = [\alpha, \tau, \lambda, D]$ are replaced by $\boldsymbol{\theta}^* = [\alpha^*, \tau^*, \lambda^*, D^*]$, the system $M(\boldsymbol{\theta})$ is said to be structurally globally identifiable (s.g.i) if $M(\boldsymbol{\theta}) = M(\boldsymbol{\theta}^*)$ implies that $\boldsymbol{\theta} = \boldsymbol{\theta}^*$.

Given the linear nature of the model (Eq.1) both in the states and the (delayed) inputs and that $\dot{\mathbf{y}}$, $\mathbf{y}$ and $\mathbf{u}$ are piecewise continuous in time and of exponential order, the Laplace transform ($\mathcal{L}[.]$) method may be used to assess whether the s.g.i. condition holds (see [1] and the references cited therein). The underlying idea is to compute the system transfer matrix $\mathbf{H}(s, \boldsymbol{\theta}) = \mathbf{Y}(s)/\mathbf{U}(s)$, with $\mathbf{Y}(s) = \mathcal{L}[\mathbf{y}(t)]$ and $\mathbf{U}(s) = \mathcal{L}[\mathbf{u}(t)]$, and to check whether:

$$\mathbf{H}(s, \boldsymbol{\theta}) = \mathbf{H}(s, \boldsymbol{\theta}^*) \quad \Leftrightarrow \quad \boldsymbol{\theta} = \boldsymbol{\theta}^*; \quad \forall s \tag{3}$$

Note that Eq. 3 results in a set of equations binding $\boldsymbol{\theta}$ and $\boldsymbol{\theta}^*$. If for almost any $\boldsymbol{\theta}$ this set of equations has a unique solution for $\boldsymbol{\theta}^*$, $M(\boldsymbol{\theta})$ is structurally globally identifiable (s.g.i.), if the set of solutions is finite, $M(\boldsymbol{\theta})$ is said to be structurally locally identifiable (s.l.i).

Since the computation is symbolic, writing the transfer matrix in canonical form will simplify the analysis. A canonical form may be obtained by writing down each entry of the transfer matrix as the ratio of two polynomials ordered in s, provided that the numerator and the denominator are simplified by their greatest common divisor and that the coefficient of the denominator monomial with highest degree is set to one.

Taking into consideration that $u(t-\tau) = u(t) * \delta(t-\tau)$, the Laplace transform of the system 1 reads:

$$s\mathbf{I}\mathbf{Y}(s) = \alpha e^{-\tau s}\mathbf{I}\mathbf{U}(s) + \mathbf{A}\mathbf{Y}(s) \tag{4}$$

by means of the Gershgorin circle theorem, for example, it is straightforward to get a sufficient condition for $[s\mathbf{I}-\mathbf{A}]$ to be invertible and assuming that $\mathbf{u}(t)gt\mathbf{0}$ for some t values (before and after mitosis), the system transfer matrix reads:

$$\mathbf{H}(s) = \alpha e^{-\tau s}[s\mathbf{I}-\mathbf{A}]^{-1} \tag{5}$$

In order to write each of the components of the transfer matrix as the ratio of two polynomials the Padé formula is used to approximate the exponential term $e^{-\tau s}$ by a rational function. For the sake of simplicity in the analysis a first order approximation will be used: $e^{-\tau s} = (2-\tau s)/(2+\tau s)$.

Exploiting the fact that the matrix $[s\mathbf{I}-\mathbf{A}]$ is tridiagonal and symmetric it is possible to analytically obtain its inverse (see, for example, [2]) and therefore the elements of the transfer matrix result:

$$H_{ij}(s) = \alpha\frac{2-\tau s}{2+\tau s}\frac{(-1)^{i+j}D^{j-1}\gamma_{i-1}\beta_{i+1}}{\gamma_n} \tag{6}$$

with:

- $\gamma_0 = 1$; $\gamma_1 = s - a_0$; $\gamma_i = (s-a_1)\gamma_{i-1} - D^2\gamma_{i-2}$, $i \geq 2$
- $\beta_n + 1 = 1$; $\beta_n = s - a_0$; $\beta_i = (s-a_1)\beta_{i+1} - D^2\beta i + 2$, $i \leq n-1$

and H_{ij} $=H_{ji}$ (the transfer matrix is symmetric).

It should be noted that, even though the matrix $[s\mathbf{I}-\mathbf{A}]$ is tridiagonal and symmetric, its inverse will be dense, thus $H_{ij} \neq \mathbf{0}, \forall i,j \leq n$. In addition the complexity of the corresponding elements increases with the size of the model, i.e. with n, due to the recursive nature of the expressions in Eq. 6.

We now limit the analysis to a simplified model with a reduced spatial domain of 3 nuclei. This will not affect general conclusions and simplifies the symbolic manipulation. The canonical form of the transfer matrix results:

$$\begin{aligned}
H_{11}(s) &= \frac{-\frac{1}{2}\tau\alpha s^3 - \frac{s^2\alpha\left(3D\tau^2+2\tau(\tau\lambda-1)\right)}{2\tau} - \frac{s\alpha\left(2D^2\tau+6D(\tau\lambda-2)+2\lambda(\tau\lambda-4)\right)}{2\tau} - \frac{\alpha\left(-2D^2\tau-6D\tau\lambda-2\tau\lambda^2\right)}{2\tau}}{s^4+\frac{s^3(4D\tau+3\tau\lambda+2)}{\tau}+\frac{s^2\left(3D^2\tau+8D\tau\lambda+8D+3\tau\lambda^2+6\lambda\right)}{\tau}+\frac{s\left(3D^2\tau\lambda+6D^2+4D\tau\lambda^2+16D\lambda+\tau\lambda^3+6\lambda^2\right)}{\tau}+\frac{6D^2\lambda+8D\lambda^2+2\lambda^3}{\tau}}\\
H_{12}(s) &= \frac{\frac{-sD\alpha+2D\alpha}{\tau}}{s^3+\frac{s^2(3D\tau+2\tau\lambda+2)}{\tau}+\frac{s\left(3D\tau\lambda+6D+\tau\lambda^2+4\lambda\right)}{\tau}+\frac{6D\lambda+2\lambda^2}{\tau}}\\
H_{13}(s) &= \frac{-sD^2\alpha+\frac{2D^2\alpha}{\tau}}{s^4+\frac{s^3(4D\tau+3\tau\lambda+2)}{\tau}+\frac{s^2\left(3D^2\tau+8D\tau\lambda+8D+3\tau\lambda^2+6\lambda\right)}{\tau}+\frac{s\left(3D^2\tau\lambda+6D^2+4D\tau\lambda^2+16D\lambda+\tau\lambda^3+6\lambda^2\right)}{\tau}+\frac{6D^2\lambda+8D\lambda^2+2\lambda^3}{\tau}}\\
H_{22}(s) &= \frac{-s^2\alpha+\frac{s2\alpha\left(-\frac{D\tau}{2}-\frac{\tau\lambda}{2}+1\right)}{\tau}+\frac{2\alpha(D+\lambda)}{\tau}}{s^3+\frac{s^2(3D\tau+2\tau\lambda+2)}{\tau}+\frac{s\left(3D\tau\lambda+6D+\tau\lambda^2+4\lambda\right)}{\tau}+\frac{6D\lambda+2\lambda^2}{\tau}}\\
H_{23}(s) &= \frac{\frac{-sD\alpha+2D\alpha}{\tau}}{s^3+\frac{s^2(3D\tau+2\tau\lambda+2)}{\tau}+\frac{s\left(3D\tau\lambda+6D+\tau\lambda^2+4\lambda\right)}{\tau}+\frac{6D\lambda+2\lambda^2}{\tau}}\\
H_{33}(s) &= \frac{-\frac{1}{2}\tau\alpha s^3 - \frac{s^2\alpha\left(3D\tau^2+2\tau(\tau\lambda-1)\right)}{2\tau} - \frac{s\alpha\left(2D^2\tau+6D(\tau\lambda-2)+2\lambda(\tau\lambda-4)\right)}{2\tau} - \frac{\alpha\left(-2D^2\tau-6D\tau\lambda-2\tau\lambda^2\right)}{2\tau}}{s^4+\frac{s^3(4D\tau+3\tau\lambda+2)}{\tau}+\frac{s^2\left(3D^2\tau+8D\tau\lambda+8D+3\tau\lambda^2+6\lambda\right)}{\tau}+\frac{s\left(3D^2\tau\lambda+6D^2+4D\tau\lambda^2+16D\lambda+\tau\lambda^3+6\lambda^2\right)}{\tau}+\frac{6D^2\lambda+8D\lambda^2+2\lambda^3}{\tau}}
\end{aligned}$$

Using symbolic manipulation it is possible to asses condition in Eq. 3 is satisfied. Therefore the model is structurally globally identifiable under a general input provided $u_i(t-\tau) \neq 0$, i.e. all parameters may be given unique solutions provided protein are available for time points before or after mitosis (*i. e.* when the production rate is not zero.

References

1. Walter E, Pronzato L (1997) Identification of parametric models from experimental data. Berlin: Springer.

2. da Fonseca C, Petronilho J (2001) Explicit inverses of some tridiagonal matrices. Linear Algebra and its Applications 325: 7 - 21.
