# Supplementary material for: Reverse-Engineering Post-Transcriptional Regulation of Gap Genes in Drosophila melanogaster
Source: PLoS Comput Biol. 2013 Oct 31;9(10):e1003281. doi: 10.1371/journal.pcbi.1003281 (PMC3814631; doi:10.1371/journal.pcbi.1003281)
Supplement: Text S3 — Comparison of global optimization solvers. (PDF) [file pcbi.1003281.s010.pdf]

Supplementary Text S3: Comparison of global optimization solvers.

In order to corroborate the robust performance and to evaluate the computational efficiency of our global optimisation algorithms, we performed a systematic comparison of the enhanced scatter search (eSS) approach with a number of standard implementations of state-of-the-art global optimisation methods in MATLAB:

- Covariance Matrix Adaptation Evolution Strategy (CMA-ES, [1]),
- Differential Evolution (DE, [2]),
- Simulated Annealing (SA, Matlab code based on the original algorithm by [3]),
- Enhanced Scatter Search (eSS, [4]).

All of these algorithms were applied to the optimisation problem posed in equation (2) of the main paper.

The results are presented in Supplementary Figure S5. Ten independent optimisation runs were performed for each method. Only the best curves for each method are shown. These curves indicate that SA converges to the global optimum very robustly, but at an increased computational cost compared to the other approaches. eSS, on the other hand, achieved the best performance, but with slightly less robustness than SA (data not shown). The performance/robustness of the other methods lie in between the two extremes represented by SA and eSS. For example, DE and CMA-ES show rather fast convergence, but poor robustness, since up to one in three runs resulted in stagnation at local solutions (not shown). From these results we conclude that the use of SA and eSS represents the best compromise between efficiency and robustness.

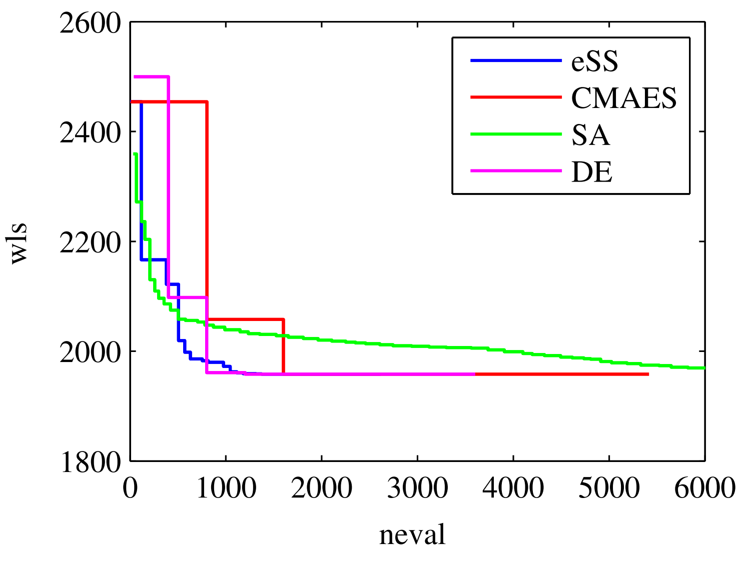

Figure S7. Convergence curves for different global optimisation approaches. Curves are shown for the *kni* model. eSS: enhanced scatter search, CMAES: covariance matrix adaptation evolution strategy, SA: simulated annealing, DE: differential evolution. 'wls' represents the weighted least squares score (equation 2 in the main text). 'neval' represents the number of model evaluations.

References

1. Hansen N (2006) The CMA evolution strategy: a comparing review. In: Lozano J, Larranaga P, Inza I, Bengoetxea E, editors, Towards a new evolutionary computation. Advances on estimation of distribution algorithms, Springer. pp. 75–102.

2. Price KV, Storn RM, Lampinen JA (2005) Differential Evolution A Practical Approach to Global Optimization. Natural Computing Series. Berlin, Germany: Springer-Verlag.

3. Kirkpatrick S, Jr DG, Vecchi MP (1983) Optimization by simulated annealing. Science 220: 671–680.

4. Egea JA, Martí R, Banga JR (2010) An evolutionary method for complex-process optimization. Comput Oper Res 37: 315–324.
