# Supplementary material for: Reverse-Engineering Post-Transcriptional Regulation of Gap Genes in Drosophila melanogaster
Source: PLoS Comput Biol. 2013 Oct 31;9(10):e1003281. doi: 10.1371/journal.pcbi.1003281 (PMC3814631; doi:10.1371/journal.pcbi.1003281)
Supplement: Text S4 — Sensitivity of parameter estimates to mRNA data. (PDF) [file pcbi.1003281.s011.pdf]

## Supplementary Text S4: Sensitivity of parameter estimates to mRNA data.

Analogous to the bootstrap analysis performed using perturbed gap gene protein data for fitting, we assessed the sensitivity of parameter estimates with respect to noise in the mRNA data (external inputs): mRNA data for the three gap genes *Kr*, *kni*, and *gt* were perturbed using values drawn from a normal distribution with mean zero and standard deviation given by the error in measurement at each data point (see Materials and Methods section of the main text, and Supplementary Figure S9A). Expression values which resulted negative after randomization were set to zero.

Resulting parameter distributions are shown in Supplementary Figure S10. All system parameters, apart from diffusion rates $D$, tend to lie within similar ranges of values compared to the bootstrap analysis using perturbed protein data (compare Supplementary Figure S10 with Figure 6 of the main paper). Values for $\alpha$ (production) and $\lambda$ (decay rates) tend to be slightly higher in this analysis. However, diffusion rate values (D) are generally much greater than estimated by bootstrapping with noisy protein data. This probably reflects an artifact of the mRNA perturbation method: considerable differences in mRNA expression levels between neighboring nuclei in perturbed data sets (see Figure S9A) need to be compensated by high diffusion, resulting in more smoothly distributed protein expression profiles. In some cases (e.g. Dkni), parameter values even saturate at the higher limit of search space, which was set to 0.3 (arrow in Figure S10H).

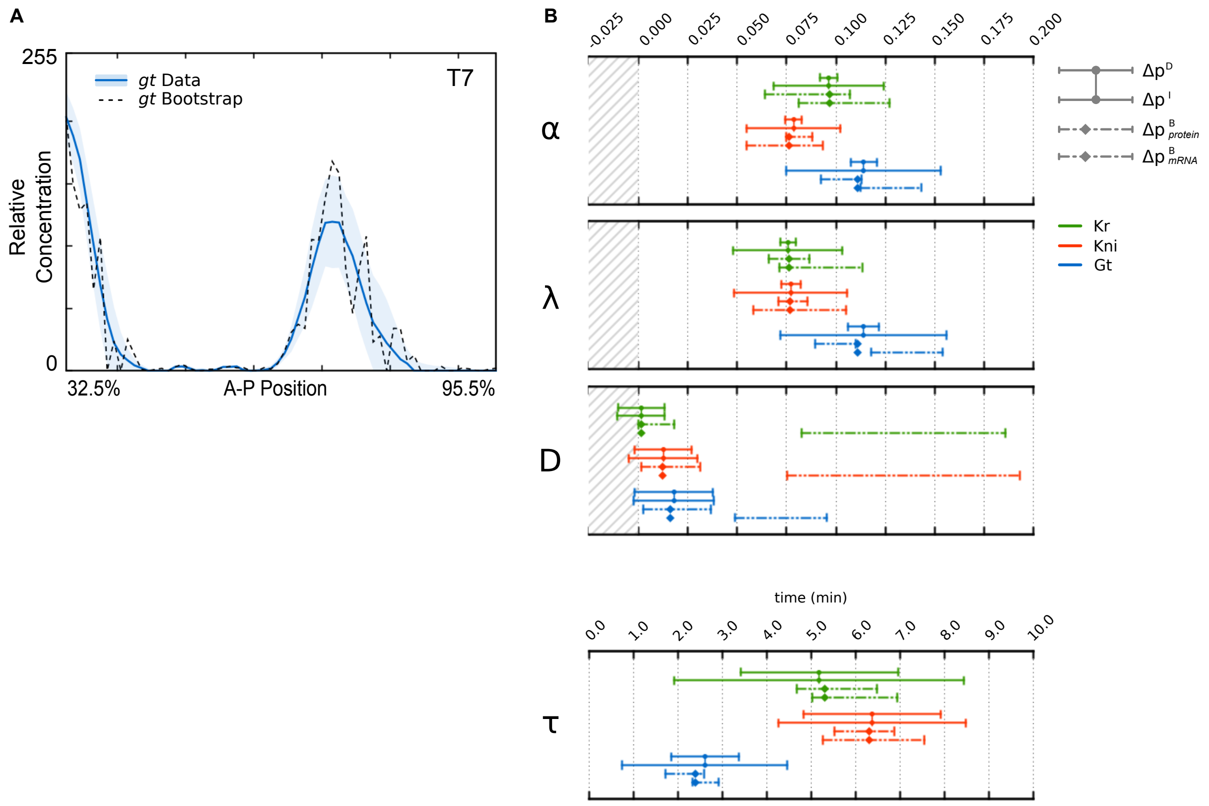


**Figure S8. Example of mRNA bootstrap data and estimated parameter confidence intervals.** (A) shows integrated *gt* mRNA data for T7 (solid line), and one standard deviation (shaded area). The dashed line depicts one (out of a 1'000) noisy bootstrap samples generated from the measured distribution of mRNA data. (B) Confidence intervals are shown as in Figure 4 of the main text. In addition, this figure shows 95%-confidence intervals as calculated from bootstrapped mRNA data.

Moreover, we see bi-clustering phenomena (similar to those shown for protein-level bootstrapping in Figure 6D of the main text) in $\tau_{kni}$ (243 parameter sets with $\tau_{kni} > 10$ min) and $\tau_{Kr}$ (84 parameter sets with $\tau_{Kr}$ ¡ 3). Therefore, we split parameter distributions into subsets based on their value of $\tau$ and calculated 95%-confidence intervals for those subsets chosen to be more biologically relevant (circles + arrows in Figure S10D and E, and confidence intervals in Figure S9B). As expected, confidence intervals for D in all three cases are significantly greater, covering higher parameter value ranges, than estimated from the protein bootstrap. As mentioned previously, this represents a smoothing artefact. In constrast, confidence intervals for production and decay rates as well as delay time are in a similar range compared to the original bootstrap analysis. This suggests that our parameter estimates are not only practically identifiable, but also robust toward perturbation of the external inputs provided by mRNA data.

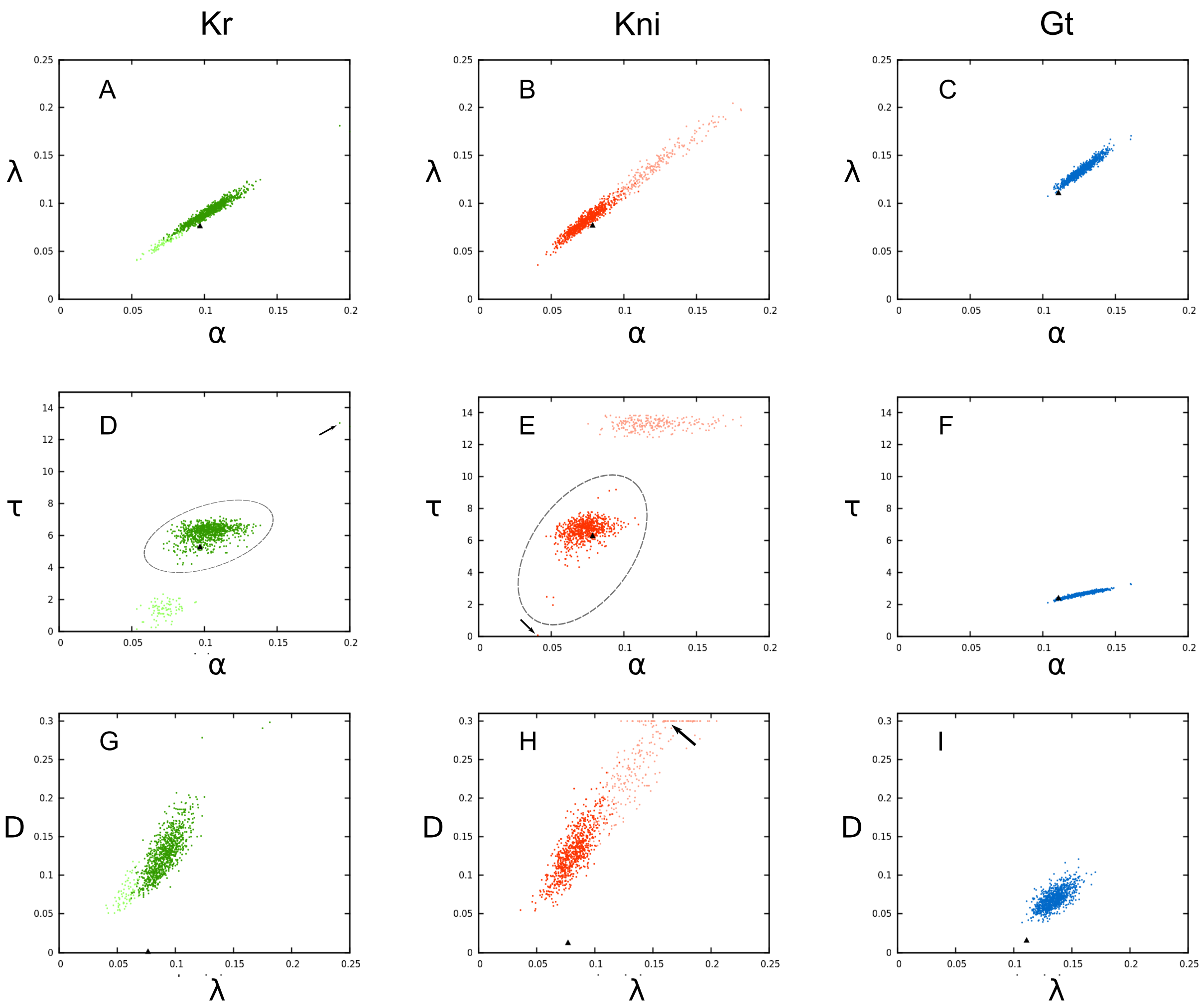


**Figure S9. Parameter distributions obtained from mRNA bootstrapping.** This Figure shows illustrative examples of scatter plots for parameter values derived from 1′000 fits to simulated noisy mRNA data (equivalent to Figure 6 in the main paper). Parameter values for *Kr* are shown in green (left column, A, D, G), for *kni* in red (center column, B, E, H), and for *gt* in blue (right column, C, F, I). Parameter notation: $\alpha$ (production rate), $\lambda$ (decay rate), $D$ (diffusion rate), and $\tau$ (production delay; see equation 1 of the main text). Black triangles indicate the original parameter estimate obtained with unperturbed data. Dashed ellipse around parameter values and also arrows pointing to individual parameter sets for *Kr* (in D) and *Kni* (in E) indicates parameters selected for further analysis. Arrow in H indicates parameter values for $D_{kni}$ which are located at the higher limit of search space.
